# TRAF4 regulates ubiquitination-modulated survivin turnover and confers radioresistance

**DOI:** 10.7150/ijbs.87180

**Published:** 2024-01-01

**Authors:** Jinzhuang Liao, Xiang Qing, Xiaoying Li, Yu Gan, Ruirui Wang, Shuangze Han, Wei Li, Wei Song

**Affiliations:** 1 Department of Radiology, The Third Xiangya Hospital of Central South University, Changsha, 410013, Hunan, China.; 2 Cell Transplantation and Gene Therapy Institute, The Third Xiangya Hospital of Central South University, Changsha, 410013, Hunan, China.; 3 Department of Otolaryngology Head and Neck Surgery, The Third Xiangya Hospital of Central South University, Changsha, 410013, Hunan, China.; 4 Hunan Provincial People's Hospital, The First Affiliated Hospital of Hunan Normal University, Changsha, Hunan, 410005, China.

**Keywords:** Nasopharyngeal carcinoma, TRAF4, Survivin, Radioresistance, Ubiquitination

## Abstract

Nasopharyngeal carcinoma (NPC) is the most common cancer originating in the nasopharynx. Despite continuous improvement in treatment strategies, recurrence or persistence of cancer after radiotherapy is still inevitable, highlighting the need to identify therapeutic resistance factors and develop effective methods for NPC treatment. Herein, we found that TRAF4 is overexpressed in NPC cells and tissues. Knockdown TRAF4 significantly increased the radiosensitivity of NPC cells, possibly by inhibiting the Akt/Wee1/CDK1 axis, thereby suppressing survivin phosphorylation and promoting its degradation by FBXL7. TRAF4 is positively correlated with p-Akt and survivin in NPC tissues. High protein levels of TRAF4 were observed in acquired radioresistant NPC cells, and knockdown of TRAF4 overcomes radioresistant *in vitro* and the xenograft mouse model. Altogether, our study highlights the TRAF4-survivin axis as a potential therapeutic target for radiosensitization in NPC.

## Introduction

Nasopharyngeal carcinoma (NPC), an invasive malignancy, is highly prevalent in Southeast and East Asia and is frequently diagnosed at the advanced stage 1. Genetic, viral, and dietary factors are implicated in the tumorigenesis of NPC 2. Intensity-modulated radiotherapy is currently the primary management for NPC because of the radiosensitivity and complicated anatomic construction 3, 4. Although clinical treatment strategies are continuously advanced, partial patients exhibit unusual radioresistance, resulting in failure in therapy 5. Hence, exploring the immanent mechanisms of radioresistance is needed to improve prognosis with advanced NPC.

Posttranslational modifications, including phosphorylation, acetylation, and ubiquitination, are crucial processes that maintain protein turnover to regulate cellular homeostasis 6. E3 ubiquitin ligases are crucial in cancer progression as they catalyze ubiquitination by transferring ubiquitin to cancer-associated proteins 7, 8. Tumor necrosis factor receptor-associated factor 4 (TRAF4), a typical ring domain E3 ubiquitin ligase 9, is frequently overexpressed and is correlated with poor prognosis in human malignancies, including colorectal 10, prostate 11, lung 12 cancer, and glioblastoma 13. TRAF4 functions as an oncoprotein to regulate metastasis 14, oncogenesis 15, and therapeutics resistance 10, 16. Multiple signalings have been demonstrated to be regulated by TRAF4, including CHK1 16, Wnt/β-catenin^17^, Smurf2 18, cAMP 11, NF-κB 9, and YAP 19. However, the oncogenic function of TRAF4 in NPC is still unclear.

Survivin, a unique inhibitor of apoptosis (IAP) protein family member, encoded by the BRIC5 gene and frequently overexpressed in human cancers 20, 21 and implicated in poor survival and disease recurrence 22, 23. Upregulation of Survivin contributes to cell cycle progression and apoptosis dysregulation, which is correlated with tumorigenesis in NPC 24-26. Survivin is conducive to maintaining EBV genomes in EBV-positive Burkitt lymphoma cells 27 and is used as a biomarker to predict the malignancy of NPC 28, 29. Therefore, targeting survivin is a promising strategy for cancer treatment.

In this study, we investigated the effect of TRAF4 on the radiosensitivity of NPC cells, finding that TRAF4/survivin signaling played a vital role in promoting the radioresistance of NPC cells. Our results provide novel insights to improve NPC radiosensitivity.

## Methods

### Reagents and antibodies

NaCl, Tris base, SDS, and DMSO were obtained from Sigma-Aldrich (St. Louis, MO). The chemicals, including MG132, cycloheximide (CHX), and MK2206, were purchased from Selleck Chemicals (Houston, TX). Necrostatin-1, z-VAD-fmk, 3-MA, Adavosertib, and Flavopiridol were products of MedChemExpress (Monmouth Junction, NJ). The cell culture media, fetal bovine serum, and antibiotics were obtained from Invitrogen (Grand Island, NY). Antibodies against Survivin (#2808; IB: 1:1000; IHC: 1: 500), p-Survivin T34 (#8888; 1:1000), α-Tubulin (#2125; 1:1000), Ub-k48 (#8081; 1:1000), Bax (#14796; 1:1000), p-Wee1 Ser642 (#4910; 1:1000), Akt (#4691; 1:1000), p-CDK1 Thr161 (#9114; 1:1000), VDAC1 (#4866; 1:1000), p-Akt Ser473 (#4060; IB: 1:1000; IHC: 1: 200), cytochrome C (#11940; 1:1000), cleaved-caspase 3 (#9664; IB: 1:1000; IHC: 1: 2000), p-CDK1 Tyr15 (#4539; 1:1000), γ-H2AX (#9718; IB: 1:1000; IF: 1: 800), cleaved-PARP (#5625; 1:1000), β-actin (#3700; 1:1000), cIAP2 (#3130; 1:1000), cIAP1 (#7065; 1:1000), and XIAP (#2045; 1:1000), Akt1 (#2938; 1:1000), Ub (#3936; 1:1000), Flag-tag (#8146; 1:1000), and anti-mouse IgG HRP (#7076; 1:10000), anti-rabbit IgG HRP (#7074; 1:10000) were obtained from Cell Signaling Technology, Inc. (Beverly, MA). Antibodies against FBXL7 (#ab59149; 1:1000) and Ki67 (#ab15580; IHC: 1: 2000) were products of Abcam (Cambridge, UK). TRAF4 (#MABC985; IB: 1:4000; IHC: 1:300) antibody was purchased from Sigma-Aldrich (St. Louis, MO).

### Cell lines and cell culture

The immortalized nasopharyngeal epithelial cell NP460 and nasopharyngeal carcinoma cell lines HNE1, HNE3, CNE2, C666-1, CNE1, SUNE1, and HK1 were obtained from the Cell Bank of Central South University, Changsha, China. HEK293T cells were obtained from the American Type Culture Collection (Manassas, VA). All cells were cultured at 37 ℃ in a humidified incubator with 5% CO_2_ under the guidance of standard protocols and detected mycoplasma infection every 2 months. The radioresistant cell lines CNE2R and HK1R were generated in our lab as previously described 30. Briefly, irradiation (2 Gy) treated CNE2 and HK1 cells consecutively to a final dose of 80 Gy. The irradiated cells were cultured at 37 °C in a humidified incubator with 5% CO_2_ and were passaged into another flask when confluency was approximately 80%. Irradiation was repeated over 6 months.

### Clinical tissue sample collections

Pathology samples, including NPC tumor tissues and the matched adjacent non-tumor tissues, were obtained from the Department of Otolaryngology at the Third Xiangya Hospital of Central South University with written informed (n = 67). Patients were diagnosed and categorized by the Departments of Pathology of the Third Xiangya Hospital under WHO protocols. None of the patients received any treatment before surgery.

### Cell viability assay

Human NPC cells were seeded at a density of 3×10^3^/well in 96-well plates and treated without or with IR for 24 h. MTS regent (#G3581, Promega, Madison, WI) was added to each well and cultured for 1.5 h at 37 ℃. Cell viability was detected under the standard protocol. Three independent experiments were carried out in triplicate.

### Soft agar assay

Soft agar assay was performed as previously described 31. Briefly, NPC cells (8 × 10^3^ cells/well) were suspended in 1 mL 0.3% agar with 10% FBS Eagle's medium and then seeded into a 6-well plate containing 0.6% agar base and maintained for 2 weeks. Images were taken and colonies were counted under a microscope.

### Immunoblotting (IB) and co-immunoprecipitation (Co-IP) assay

The immunoblotting and co-immunoprecipitation assays were conducted as described previously 32. Proteins were harvested by RIPA buffer with protease inhibitors. The protein concentration was determined using the BCA protein assay kit. The proteins were separated using a Bolt 4-20% Gel. For the Co-IP assay, the IP buffer was used to collect the cell lysates, and then the cell lysate was hybridized with the indicated antibody and Protein A/G agarose beads. After washing the beads with ice-cold PBS, 2X SDS-PAGE loading buffer (40 µL) was added to resuspend the beads and boiled for 5 min. The target protein contained supernatant was separated utilizing SDS-PAGE electrophoresis by transferring the proteins to polyvinylidene difluoride membranes. Following blocking with BSA for 1 h, the membranes were incubated with the primary antibody overnight at 4 ˚C, and then the indicated second antibody for 30 min at room temperature (RT). The interested protein was visualized utilizing the enhanced chemiluminescence reagents.

### Plate colony formation assay

NPC cells were treated with IR and culture for 24 h, then the cells were counted, and 500 cells were seeded into a 6-well plate and incubated for 2 weeks until visible colonies appeared on the plate. The 4% paraformaldehyde was used to fix the colonies for 15 min, followed by staining the colonies for 3 min using 0.5% crystal violet at RT. The colonies were counted under a microscope.

### Plasmid construction

His-Ub (#31815) was the product of Addgene (Watertown, MA, USA). Flag-TRAF4 (#RC200345) and Flag-Survivin (#RC205935) were purchased from OriGene Technologies, Inc. Flag-TRAF4 (C18A), Flag-Survivin (T34A) and Flag-Survivin (T34D) mutant were established using the Q5 Site-Directed Mutagenesis Kit (cat. #E0554S; New England BioLabs, Inc.) following the manufacturer's protocols and validated using Sanger DNA sequencing as described previously 16, 33.

### siRNA

The siCtrl (sc-37,007), survivin siRNA (sc-29499), XIAP siRNA (sc-37508), and FBXL7 siRNA (sc-62,306) were obtained from Santa Cruz Biotechnology (Dalla, TX). siRNA transfection was performed according to the manufacturer's protocol. Briefly, cells were grown to 60-80% confluence and cultured with antibiotic‐ and serum‐free medium. Add the siRNA duplexes solution directly to the dilute transfection reagent, followed by mixing and incubating for 20 min at RT. The mixture was added to cells in antibiotic‐ and serum‐free medium. After 5-7 h incubation, cells were incubated with 10% FBS and 1% P/S for 24 h.

### Generation of TRAF4 knockdown stable cell lines

Transient transfection reagent Lipofectamine™ 2000 (#11668019) and pLKO.1-shTRAF4 lentivirus plasmids (#1, TRCN0000034242, #2, TRCN0000034241) were products of Thermo Fisher Scientific, Inc. The pLKO.1-shTRAF4 lentivirus plasmids and PMD2-G and PSPAX2 plasmids were co-transfected into 293T cells. After 72 h, the viral supernatant fractions were collected and subsequently infected the NPC cells for 24 h with polybrene (5 μg/mL). The cell medium was replaced with fresh medium containing puromycin (2 μg/mL) one day later and maintained for 1 week for stable cell selection.

### Immunofluorescence (IF)

NPC cells were exposed to IR (2 Gy) and cultured for 24 h. The cells were fixed with 4% paraformaldehyde at RT for 15 min, then permeabilized in 0.5% Triton X-100 for 10 min. Following 1 h of blocking with 10% goat serum albumin in PBS, the cells were incubated with the indicated antibody against γ-H2AX overnight at 4 ˚C and then the corresponding secondary antibody for 30 min at RT. After staining the cellular nucleus with DAPI, the images were captured using a confocal fluorescence microscope (Nikon C1si; Nikon Corporation).

### Immunohistochemical (IHC) staining

IHC staining of tissues from the mice xenograft or NPC tumor was performed as previously described 34. Tissue slides were dewaxed and rehydrated in gradient ethanol by subsequent submersion into sodium citrate buffer (10 mM, pH 6.0) and boiling for 10 min to complete antigen retrieval. The slides were incubated with 3% H_2_O_2_ for 10 min to inactivate the intrinsic horseradish peroxidase after washing with ddH_2_O for 3 times. The goat serum albumin in PBS (50%) was added to the sections, followed by incubation at RT for 1 h, and hybridized with the primary antibody in a humidified chamber overnight at 4°C. The sections were incubated with corresponding secondary antibody at RT for 45 min, visualized using DAB substrate, and then counterstained with hematoxylin.

### Ubiquitination assay

Ubiquitination assay was performed as described previously 35. The indicated plasmids were used to co-transfect cells by subsequent harvest. The cells were lysed with Ni-NTA lysis buffer (6 M guanidine-HCl, 10 mM β-mercaptoethanol, 5 mM imidazole, pH 8.0, 0.01 M Tris/HCl, and 0.1 M Na_2_HPO_4_/NaH_2_PO_4_) supplemented with protease inhibitors and 10 mM N-ethylmaleimide (NEM) for Ni-NTA pull-down assay; and with modified RIPA buffer supplemented with NEM (10 mM) and protease inhibitors for IP-mediated ubiquitination assay. The ubiquitination levels of protein were detected using IB.

### *In vivo* tumor growth

All animal experiments were approved by the Institutional Animal Care and Use Committee, the Third Xiangya Hospital of Central South University (Changsha, China). CNE2 or CNE2R (2×10^6^) cells were s.c.injected into the right flank of 6-week-old athymic nude mice (n=5) to generate the NPC xenograft models. The tumor-bearing mice were randomly divided into groups and initiated with various treatments when the tumor volume reached ~100 mm^3^. Mice (n= 5/6) were exposed to local IR by X-RAD 320 (Precision X-ray, Inc.) irradiating X-rays. For Akt inhibitor MK2206 treatment, the mice were randomly allocated to four groups (n = 5): 1, vehicle control (0.5% dimethyl sulfoxide, 100 µL/every 2 days, i.p.); 2, local IR (2 Gy, three times); 3, MK2206 (40 mg/kg/ every two days, i.p.); 4, local IR (2 Gy, three times) + MK2206 (40 mg/kg/ every two days, i.p.). The tumor volume was measured every other day and calculated by the formula perpendicular (0.5 x length x width^2^). Finally, mice were euthanized, and the tumor mass was extracted to record weight and then subjected to IHC staining.

### Statistical analysis

All Data were presented as means ± SEM from three independent experiments. All statistical analyses were performed by GraphPad Prism 5 (GraphPad 5.0, San Diego, CA, USA) or SPSS for Windows version 16.0 (SPSS, Chicago, USA). The difference between means was evaluated by one-way ANOVA or Student's t-test. Categorical data were analyzed using the Fisher exact test or χ^2^ test to assess the clinicopathologic significance of clinical samples. If the data did not fit the normal distribution, Mann-Whitney U test was done. Correlation tests were conducted using the Pearson rank correlation. Wilcoxon matched-pairs signed-rank test was used to assess the expression level difference between tumor tissues and their adjacent tissues. P < 0.05 was considered statistically significant.

## Results

### TRAF4 is required for maintaining the oncogenic capability of NPC cells

**Figure 1:**
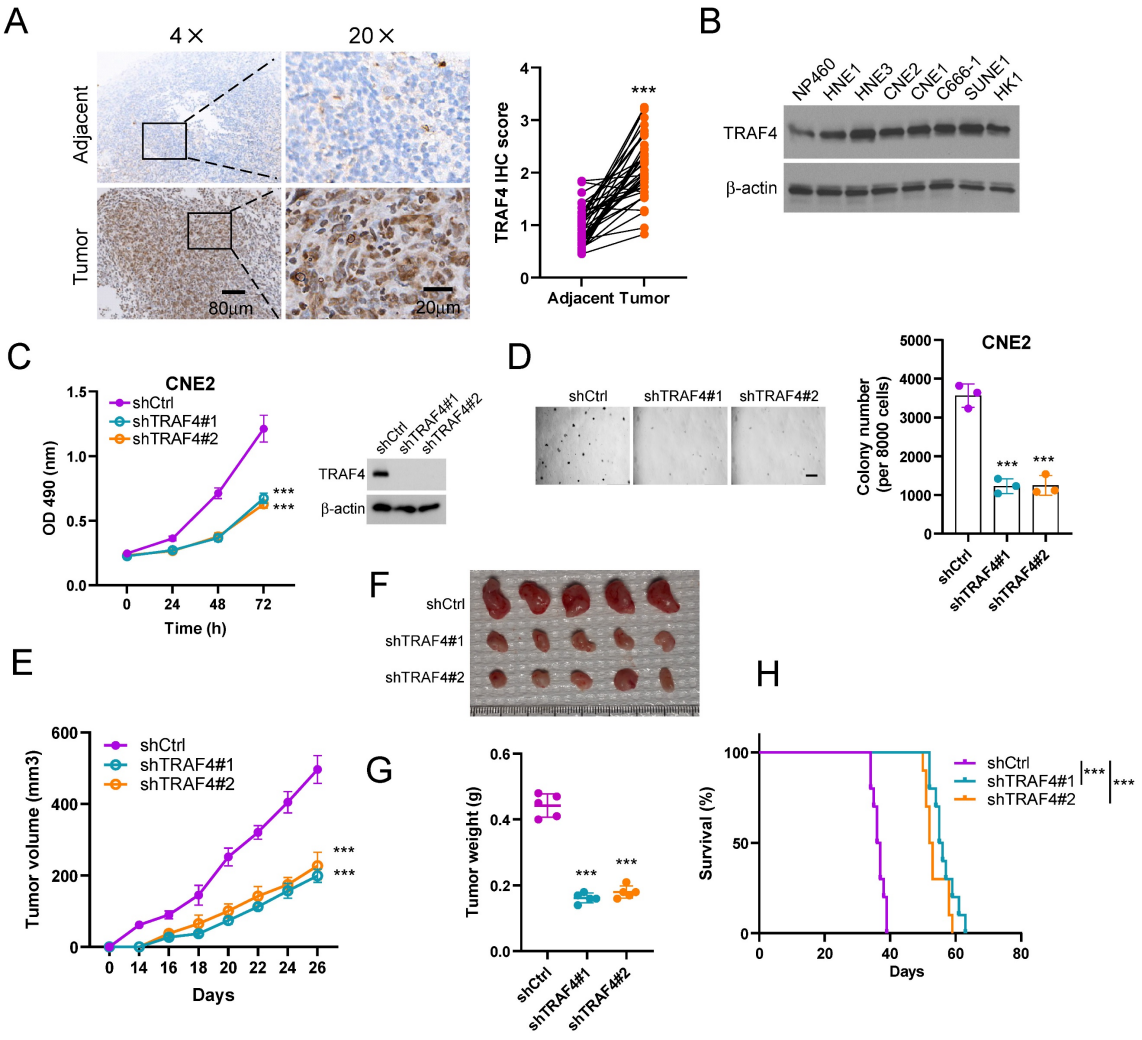
TRAF4 maintains the tumorigenic capacity of nasopharyngeal carcinoma (NPC) cells. (A) IHC analysis of TRAF4 in tumors and matched adjacent non-tumor tissues from 37 NPC patients. Scale bar 80 μm (left), and 20 μm (right), respectively. ***p < 0.001. (B) The expression level of TRAF4 was tested in immortalized nasopharyngeal epithelial cells NP460 and NPC cell lines HNE1, HNE3, CNE2, CNE1, C666-1, SUNE1, HK1 by Immunoblotting (IB) assay. (C) MTS assay (left) was performed to detect cell viability. ***p < 0.001. TRAF4 expression was validated by IB assay (right) in shTRAF4 CNE2 cells. (D) Soft agar assay analysis of colony formation of shTRAF4 CNE2 cells. Left, representative image; right, qualification. Scale bar 200 μm. ***p < 0.001. (E-G) The *in vivo* tumorigenesis of CNE2 shTRAF4 cells. E, tumor growth curve; F, tumor images; G, tumor weight. Scale bar 1 cm. ***p < 0.001. (H) The survival analysis of tumor-bearing mice with shCtrl- or shTRAF4-CNE2 xenograft tumors by Kaplan-Meier method. n=10 mice per group. ***p < 0.001.

We first determined the expression of TRAF4 in NPC tissues by IHC staining analysis. The data indicated that TRAF4 was overexpressed in NPC tissues when compared to the adjacent non-tumor tissues (Fig. 1A). Similarly, high protein levels of TRAF4 were observed in NPC cells but not that in the immortalized nasopharyngeal epithelial cell NP460 (Fig. 1B). Stable TRAF4-knockdown cell lines CNE2, HNE3, and HK1 were further established (Fig. 1C, S1A-B) with two shRNAs and the malignant phenotypes were examined. The result revealed that the cell viability and colony formation in soft agar of CNE2, HNE3, and HK1 cells were suppressed due to the depletion of TRAF4 (Fig. 1C-D and S1A-D). Next, the impact of TRAF4 on *in vivo* tumorigenesis of NPC cells was detected. The data indicated that tumor volume (Fig. 1E), mass (Fig. 1F), and weight (Fig. 1G) were significantly reduced in TRAF4-depleted CNE2 tumors. In addition, the tumor-bearing mice with TRAF4-depleted NPC cells have a more extended survival period (Fig. 1H). Our results suggest that TRAF4 is required for maintaining the tumorigenesis properties of the NPC cells.

### TRAF4 knockdown facilitates IR-activated endogenous apoptosis in NPC cells

**Figure 2:**
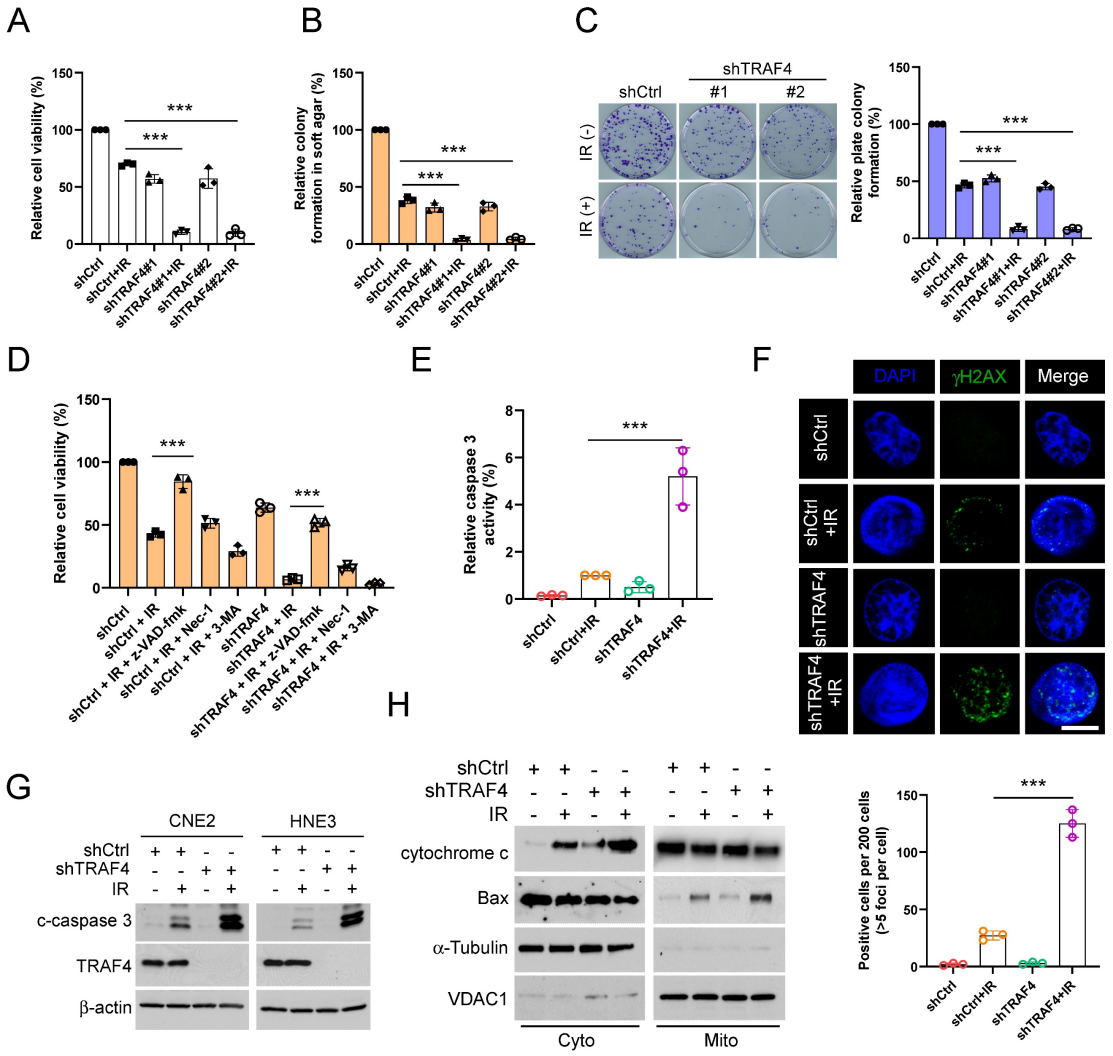
Depletion of TRAF4 elevated the sensitivity of NPC cells to irradiation (IR). (A) The cell viability of shTRAF4-CNE2 cells without or with IR (2 Gy) for 24 h was measured using MTS assay. ***p < 0.001. (B) Soft agar assay analysis of colony formation in shTRAF4 CNE2 cells in the presence of IR (2 Gy). ***p < 0.001. (C) Plate colony formation of shTRAF4 CNE2 cells in the presence or absence of IR (2 Gy). ***p < 0.001. (D) CNE2 shTRAF4 cells were pretreated with inhibitors such as z-VAD-fmk, necrostatin-1 (Nec-1), and 3-MA for 4 h, followed by IR (4 Gy) treatment and maintained for 24 h. MTS analysis was used to test cell viability. ***p < 0.001. (E-H) CNE2 shTRAF4 cells were exposed to IR (4 Gy) for 48 h. (E) The caspase 3 activity was detected using the caspase 3 assay Kit. ***p < 0.001. (F) Immunofluorescent was used to examine the expression of γ-H2AX. Top, representative images; bottom, qualification. Scale bar, 5 μm. ***p < 0.001. (G) The cleaved-caspase 3 protein level was detected using IB assay. (H) Subcellular fractions were isolated and subjected to IB analysis.

To determine whether TRAF4 impacts the sensitivity of NPC cells to radiotherapy, we analyzed the cell viability of TRAF4-depleted CNE2 and HNE3 cells with irradiation (IR). We found that IR treatment significantly inhibited cell viability (Fig. 2A and S2A), anchorage-independent (Fig. 2B and S2B) and -dependent (Fig. 2C and S2C) colony after TRAF4 depletion. To determine which cell death pathway was activated in IR-treated NPC cells, we utilized three inhibitors, including z-VAD-fmk, necrostatin-1 (Nec-1), and 3-MA, to pretreat NPC cells. These three inhibitors act on apoptosis, necroptosis, and autophagy signal pathways. As shown in Fig. 2D and S2D, apoptosis inhibitor z-VAD-fmk restored the cell viability of CNE2 and HNE3 cells with IR treatment most significantly, revealing that activation of apoptosis signal was responsible for IR-triggered NPC cell death. Moreover, the relative caspase 3 activity (Fig. 2E and S2E) was significantly increased in TRAF4-depleted cells after IR treatment. In the immunofluorescence analysis, the γ-H2AX protein, a DNA damage marker, was upregulated in TRAF4-depleted cells with IR treatment (Fig. 2F). IB data showed that irradiation increased the expression of cleaved-caspase 3 protein in TRAF4 knockdown cells after IR treatment (Fig. 2G). Subcellular fractions from CNE2 cells detected by IB assay indicated that irradiation promoted cytochrome c release to the cytoplasm and translocated Bax from the cytoplasm to mitochondria, and these effects were further strengthened with TRAF4 depletion (Fig. 2H). These results revealed that TRAF4 depletion facilitates IR-triggered endogenous apoptosis in NPC cells.

### Downregulation of survivin promotes IR-triggered apoptosis in TRAF4-depleted NPC cells

**Figure 3:**
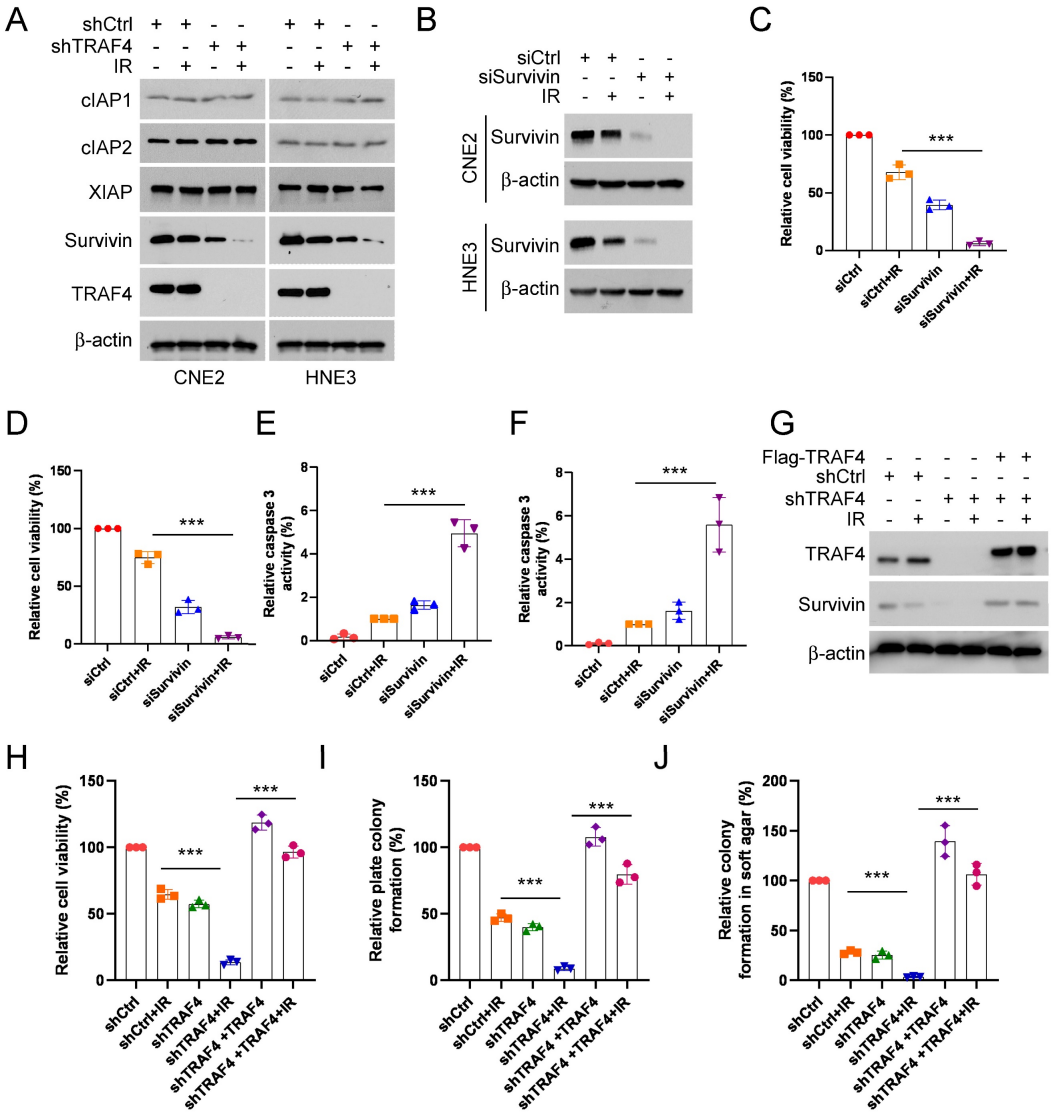
Depletion of TRAF4 impaired survivin expression. (A) CNE2 and HNE3 cells with TRAF4 depletion were treated without/with IR (2 Gy) and cultured for 24 h followed by IB assay. (B-F) CNE2 and HNE3 cells were transfected with siRNA targeting survivin for 24 h, followed by IR (2 Gy) treated and cultured for 24 h. (B) IB was conducted to detect survivin expression. MTS analysis was used to test the cell viability of CNE2 (C) and HNE3 (D) cells. ***p < 0.001. The caspase 3 activity in CNE2 (E) and HNE3 (F) cells was detected using the caspase 3 assay Kit. ***p < 0.001. (G-J) Flag-TRAF4 was transfected into CNE2 cells with TRAF4 knockdown for 24 h, followed by IR (2 Gy) treatment and cultured for 24 h. (G) The indicated proteins were measured by IB analysis. The cell viability (H), plate colony formation (I), and colony formation in soft agar (J) were examined. ***p < 0.001.

To understand the underlying mechanism of IR-triggered apoptosis, we tested the protein levels of the IAP family members, which play crucial roles in apoptosis, in shTRAF4-CNE2 and -HNE3 cells. IB data suggested that the survivin protein level was reduced in the presence of IR and was further strengthened following the depletion of TRAF4 (Fig. 3A). We next transfected siRNA into CNE2 and HNE3 cells to establish survivin-silenced cell lines and found that survivin protein level (Fig. 3B) and cell viability (Fig. 3C-D) was attenuated particularly after treatment with IR in survivin-silenced cells. Furthermore, the caspase 3 activity was elevated significantly in survivin-silenced cells following IR treatment (Fig. 3E-F). To further determine whether TRAF4 is required for maintaining survivin expression in NPC cells, we transfected Flag-TRAF4 into TRAF4 knockdown NPC cells. The results showed that overexpression of TRAF4 restored survivin protein levels (Fig. 3G), cell viability (Fig. 3H), and the ability to form colonies on plates (Fig. 3I) and in soft agar (Fig. 3J), even after being exposed to irradiation. Next, Flag-TRAF4 WT or Flag-TRAF4 C18A mutant (the catalytic inactivation mutant) was ectopically overexpressed in CNE2 and HNE3 cells. The IB data showed that Flag-TRAF4 WT but not C18A mutant promoted survivin expression (Fig. S3A). These data revealed that downregulation of survivin was required for IR-triggered apoptosis in shTRAF4-NPC cells.

### TRAF4 deficiency destabilizes survivin in a Thr34 phosphorylation-dependent manner

**Figure 4:**
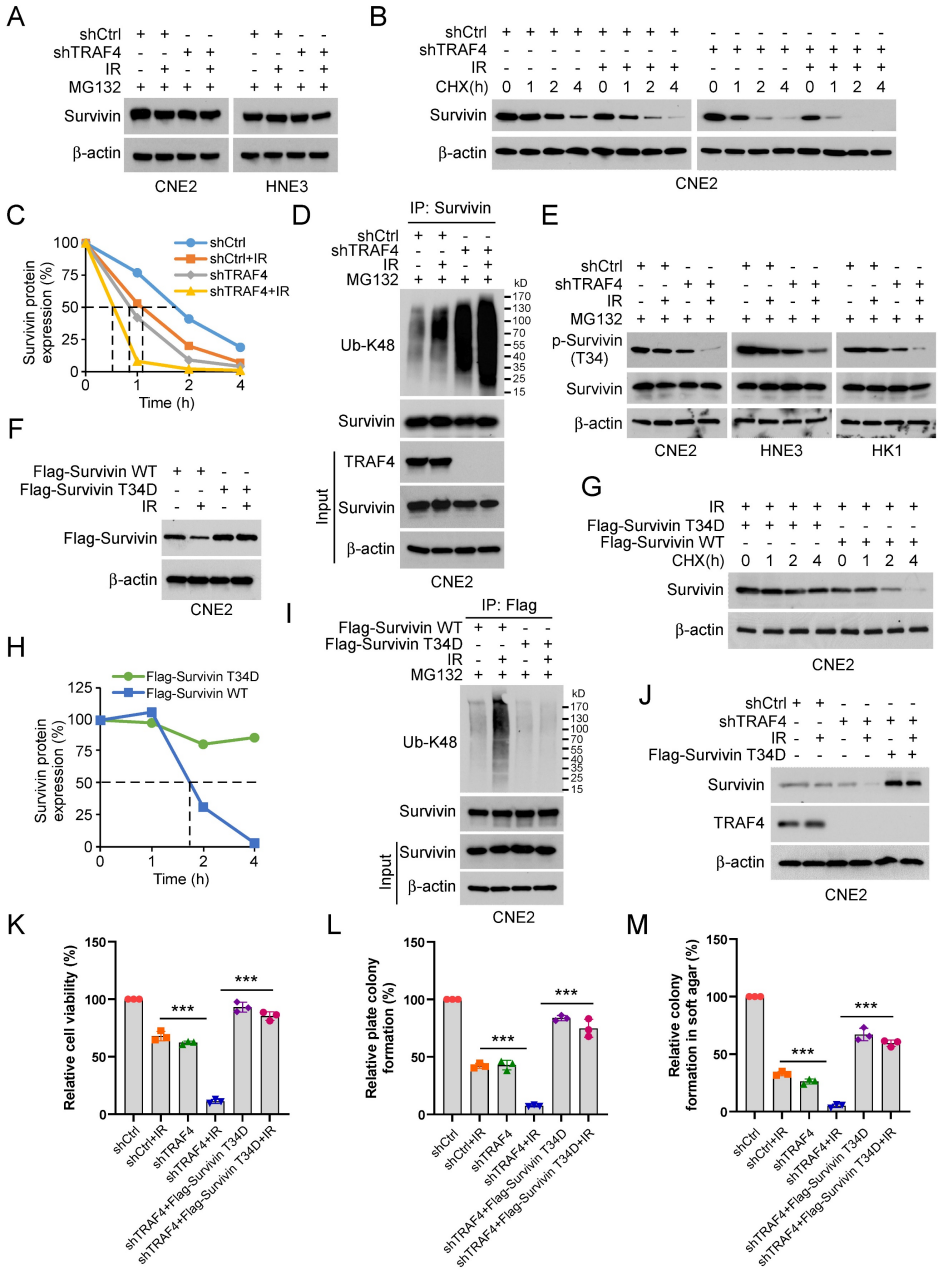
Irradiation promotes survivin degradation in TRAF4 knockdown NPC cells. (A) TRAF4-depleted CNE2 and HNE3 cells were treated with IR (2 Gy) for 24 h, then treated with MG132 (20 µM) for 6 h, and whole cell extract (WCE) was subjected to IB analysis. (B and C) IR (2 Gy) treated TRAF4-depleted CNE2 cells by subsequent incubation with CHX (20 µg/mL) at various times (B). (C) Qualification for (B). (D) Immunoprecipitation (IP) assay for survivin ubiquitination level in TRAF4 knockdown CNE2 cells treated with IR (2 Gy) for 24 h by subsequent MG132 treatment for 6 h. (E) IB for indicated protein expression in CNE2, HNE3, and HK1 cells was treated with IR (2 Gy) for 24 h, followed by treated MG132 for 6 h. (F) Flag-Survivin-WT or T34D was transfected into CNE2 cells for 24 h, then treated with IR (2 Gy) for 24 h. IB assay was performed to analyze survivin expression. (G) Various plasmids were transfected into CNE2 cells for 24 h, then treated with IR (2 Gy) for 24 h, followed by incubation with CHX for various time points. The WCE was subjected to IB analysis. (H) Qualification for (G). (I) CNE2 cells were transfected with different plasmids for 24 h, then treated with IR (2 Gy) for 24 h by subsequent MG132 treatment for 6 h. The WCE was subjected to ubiquitination assay. (J-M) Flag-Survivin-T34D was transfected into TRAF4-depleted CNE2 cells for 24 h, followed by IR (2 Gy) treatment and cultured for 24 h. WCE was subjected to IB analysis (J), cell viability was examined by MTS assay (K), and colony formation was tested by plate colony formation (L) and soft agar assay (M). ***p < 0.001.

To investigate the mechanism of how survivin was downregulated in TRAF4-depleted NPC cells in the presence of IR, TRAF4-depleted CNE2 and HNE3 cells were treated with MG132, a proteasome inhibitor following IR treatment. The IB data suggested that the endogenous survivin protein was stabilized after MG132 treatment (Fig. 4A). Next, we performed the cycloheximide (CHX) protein assay to determine whether TRAF4 impacted survivin stability in TRAF4 knockdown CNE2 stable cells. The data indicated that both TRAF4 deficiency and IR treatment shortened the half-life of survivin and further enhanced in combination (Fig. 4B-C). These data revealed that the downregulation of survivin is likely caused by the ubiquitin-26S proteasomal degradation pathway in TRAF4 knockdown CNE2 cells after IR treatment. Therefore, we performed the ubiquitination assay and found that survivin K48-linked polyubiquitination was elevated prominently in TRAF4 knockdown CNE2 cells following IR treatment (Fig. 4D). Phosphorylation of survivin on Thr34 is essential for maintaining its stability. Hence, we investigated the effect of IR on the phosphorylation of survivin in TRAF4-depleted CNE2, HNE3, and HK1 cells. As shown in Fig. 4E, the phosphorylation of survivin (Thr 34) was decreased. We constructed a survivin T34D mutant, in which the Thr 34 was mutated to aspartic acid (T34D) to mimic its phosphorylation. The IB data suggested that the survivin (T34D) protein level was unaffected after irradiation (Fig. 4F). Furthermore, the T34D mutation extended the half-life of survivin ​(Fig. 4G-H). The IB data indicated that T34D mutation substantially reduced survivin ubiquitination following IR treatment (Fig. 4I). Flag-Survivin-T34D was used to overexpress in TRAF4-depleted CNE2 cells ectopically. The data showed that Survivin-T34D restored survivin protein levels (Fig. 4J), cell viability (Fig. 4K), and the ability to form colonies on plates (Fig. 4L) or in soft agar (Fig. 4M), even when exposed to irradiation. We next transfected Flag-Survivin WT into shTRAF4-CNE2 cells. The results showed that Flag-Survivin WT reversed the reduction of survivin protein levels (Fig. S4A), cell viability (Fig. S4B), the ability to form colonies on the plates (Fig. S4C) and in soft agar (Fig. S4D) of shTRAF4-CNE2 cells with IR treatment. Collectively, our results show that TRAF4 deficiency facilitated the reduction of IR-induced survivin phosphorylation and stability.

### Suppression of Akt/Wee1 signal caused survivin degradation in TRAF4 knockdown cells

**Figure 5:**
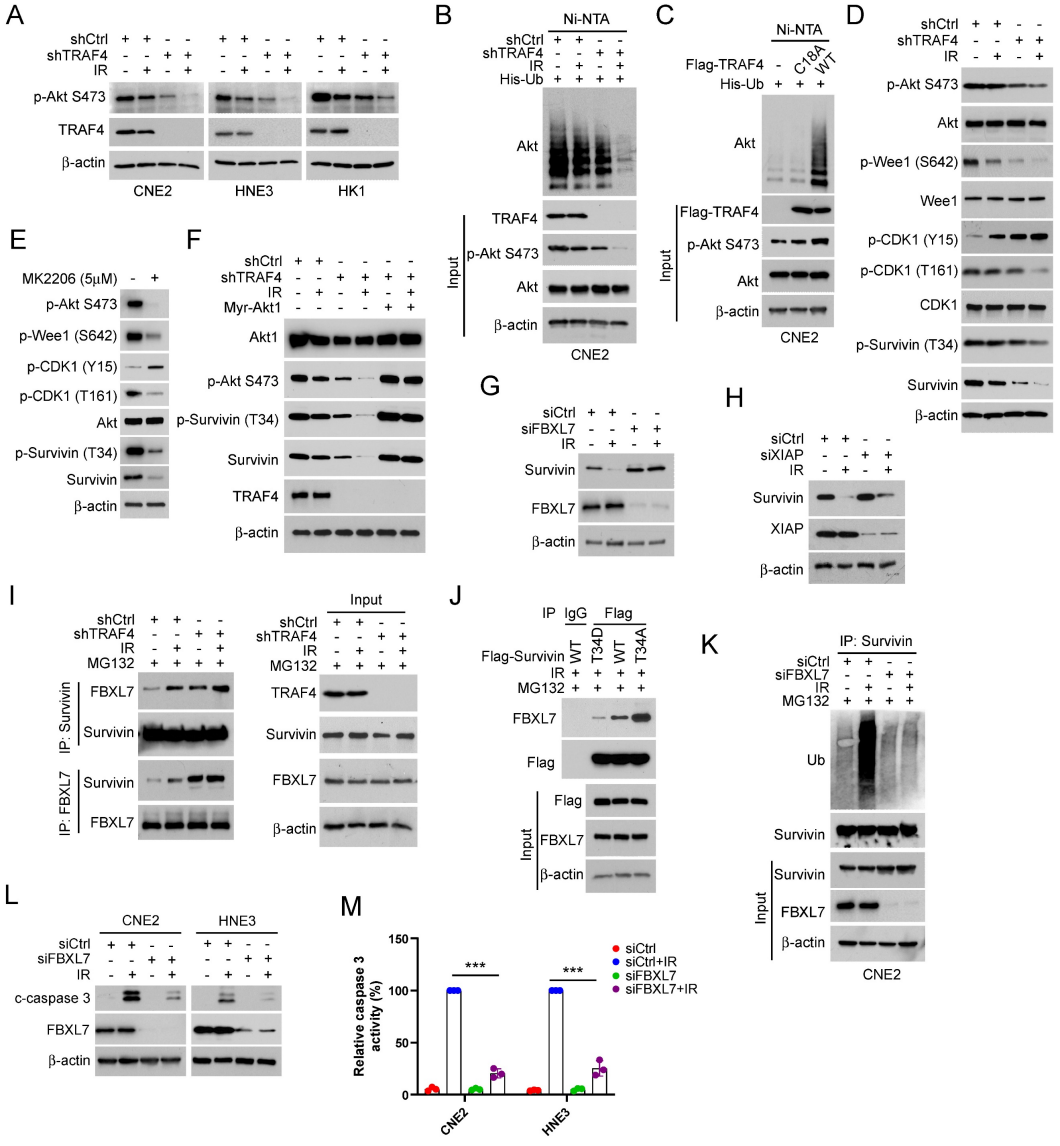
TRAF4-regulated Akt/Wee1 signaling is required for survivin stabilization. (A) TRAF4 knockdown NPC stable cells were exposed to IR (2 Gy) for 24 h, followed by IB analysis. (B) His-Ub was transfected into CNE2 shCtrl and shTRAF4 cells for 24 h, then treated with IR (2 Gy) for 24 h. Akt ubiquitination was detected by Ni-NTA pull-down assay. WCE was subjected to IB analysis. (C) Flag-TRAF4 WT or C18A mutant and His-Ub were transfected into CNE2 cells for 24 h. Ni-NTA pull-down assay was conducted to measure the ubiquitination of Akt. (D) IB analysis of TRAF4 knockdown CNE2 cells exposed to IR (2 Gy) or not for 24 h. (E) CNE2 cells were treated with MK2206 (5µM) for 24 h, the WCE was subjected to IB assay. (F) Mry-Akt1 was transfected into TRAF4-depleted CNE2 cells for 24 h, followed by IR (2 Gy) for another 24 h. WCE was subjected to IB analysis. (G and H) CNE2 cells were transfected with siCtrl, siFBXL7 (G) or siXIAP (H) for 24 h. Cells were exposed to IR (2 Gy) for 24 h. WCE was collected and subjected to IB assay. (I) CNE2 shCtrl and shTRAF4 cells were treated with IR (2Gy) for 24 h by subsequent MG132 treatment for 6 h, WCE was collected and subjected to IP assay with FBXL7 or survivin antibody, and the interaction between FBXL7 and survivin was tested by IB analysis. (J) Flag-Survivin WT, T34D, or T34A was transfected into CNE2 cells for 24 h, and then treated with IR (2Gy) for 24 h by subsequent MG132 treatment for 6 h. WCE was subjected to Co-IP and IB analysis. (K-M) The siCtrl or siFBXL7 was transfected into CNE2 cells followed by treatment without/with IR (2 Gy) for 24 h, survivin ubiquitination assay was examined (K), cleaved-caspase 3 was examined by IB analysis (L). The caspase 3 assay Kit was used to detect the caspase 3 activity (M). ***p < 0.001.

IB assay was conducted to determine whether TRAF4 affects Akt signaling in IR-treated NPC cells. The results showed that Akt phosphorylation on Ser473 was reduced prominently in shTRAF4-CNE2, -HNE3, and -HK1 cells, especially in the presence of IR for 24 h (Fig. 5A). K63-ubiquitination mediated by TRAF4 plays a crucial role in EGF-induced Akt activation 36. Our data showed that the ubiquitination of Akt was decreased in shTRAF4-CNE2 cells after IR treatment (Fig. 5B). TRAF4 wild-type (WT), but not C18A mutant, promoted Akt polyubiquitination and phosphorylation in CNE2 cells (Fig. 5C). These results suggest that TRAF4 is required for Akt activation in NPC cells. The IB results showed that depleting TRAF4 decreased Wee1 phosphorylation at Ser642, a downstream target of Akt kinase, in CNE2 cells with IR treatment. Moreover, depleting TRAF4 enhanced the phosphorylation of CDK1 on Tyr15 and decreased the phosphorylation of CDK1 on Thr161 and survivin on Thr34, and total survivin level in CNE2 cells following IR, but had no apparent impaction for total Wee1 and CDK1 levels (Fig.5D). Furthermore, treated with Akt inhibitor, MK2206, suppressed total survivin level and the phosphorylation of Wee1 (Ser642), CDK1 (Thr161), and survivin (Thr34) (Fig.5E), but MG132 treatment restored total survivin level (Fig. S5A). Similarly, MK2206 decreased survivin phosphorylation in TRAF4-overexpressed CNE2 cells (Fig. S5B). To further determine the effect of Akt signaling on survivin in NPC cells, constitutively activated Akt, Myr-Akt1 was ectopically overexpressed. The IB data showed that the overexpression of Akt rescued IR-reduced survivin (Thr34) phosphorylation and expression in shTRAF4-CNE2 cells (Fig. 5F), and the downregulation of total survivin protein was conversed after MG132 treatment in shTRAF4-CNE2 cells (Fig. S5C). Consistently, cell viability (Fig. S5D) and the ability to form colonies on the plates (Fig. S5E) or in soft agar (Fig. S5F) was restored in Myr-Akt1 transfected CNE2 cells with TRAF4 depletion, even when subjected to IR treatment. Research reported that survivin also regulated the protein degradation of Wee1 and the phosphorylation of CDK1 37. Thus, we transfected siWee1 into CNE2 cells or treated CNE2 cells with CDK1 inhibitor (Flavopiridol) to determine whether the changes observed in Wee1 phosphorylation and CDK1 phosphorylation are a cause or consequence of Survivin phosphorylation. The results indicated that the phosphorylation of survivin was also attenuated in Wee1-silenced or Flavopiridol-treated CNE2 cells (Fig. S5G-H), indicating that survivin is a downstream target of Wee1-CDK1 signaling. The above findings reveal that TRAF4 is a crucial factor for maintaining Akt-Wee1-CDK1-survivin signaling in NPC cells. Previous studies have found that survivin is a substrate of the E3 ligase FBXL7 and XIAP 33, 38, and phosphorylation of Thr34 is required for maintaining survivin stability and avoiding proteasome-mediated degradation. We, therefore, transfected siRNA targeting FBXL7 and XIAP to CNE2 cells and found that restoration of survivin protein only in FBXL7-silenced (Fig. 5G), but not XIAP-silenced (Fig. 5H) CNE2 cells following IR treatment, indicating that FBXL7 play a crucial role in IR-reduced survivin expression in NPC cells. Moreover, the interaction between FBXL7 and survivin was strikingly enhanced in TRAF4-deficient cells, particularly when exposed to IR (Fig. 5I). As shown in Fig.5J, the interaction between Flag-Survivin WT and FBXL7 was weaker than Flag-Survivin T34A but stronger than Flag-Survivin T34D, indicating that the phosphorylation of Thr34 inhibits the interaction between survivin and E3 ligase FBXL7. Moreover, the IP-mediated ubiquitination assay data showed that FBXL7-mediated survivin ubiquitination was attenuated in FBXL7-silenced CNE2 cells even when exposed to IR (Fig. 5K). In addition, the cleaved-caspase 3 protein level (Fig. 5L) and caspase 3 activity (Fig. 5M) were reduced in FBXL7-silenced CNE2 and HNE3 cells after IR treatment, suggesting that FBXL7-mediated survivin degradation is required for IR-induced apoptosis. These data imply that suppression of the Akt/Wee1 signal caused survivin degradation in TRAF4 knockdown cells.

### Knockdown TRAF4 sensitizes NPC cells to IR treatment *in vivo*

**Figure 6:**
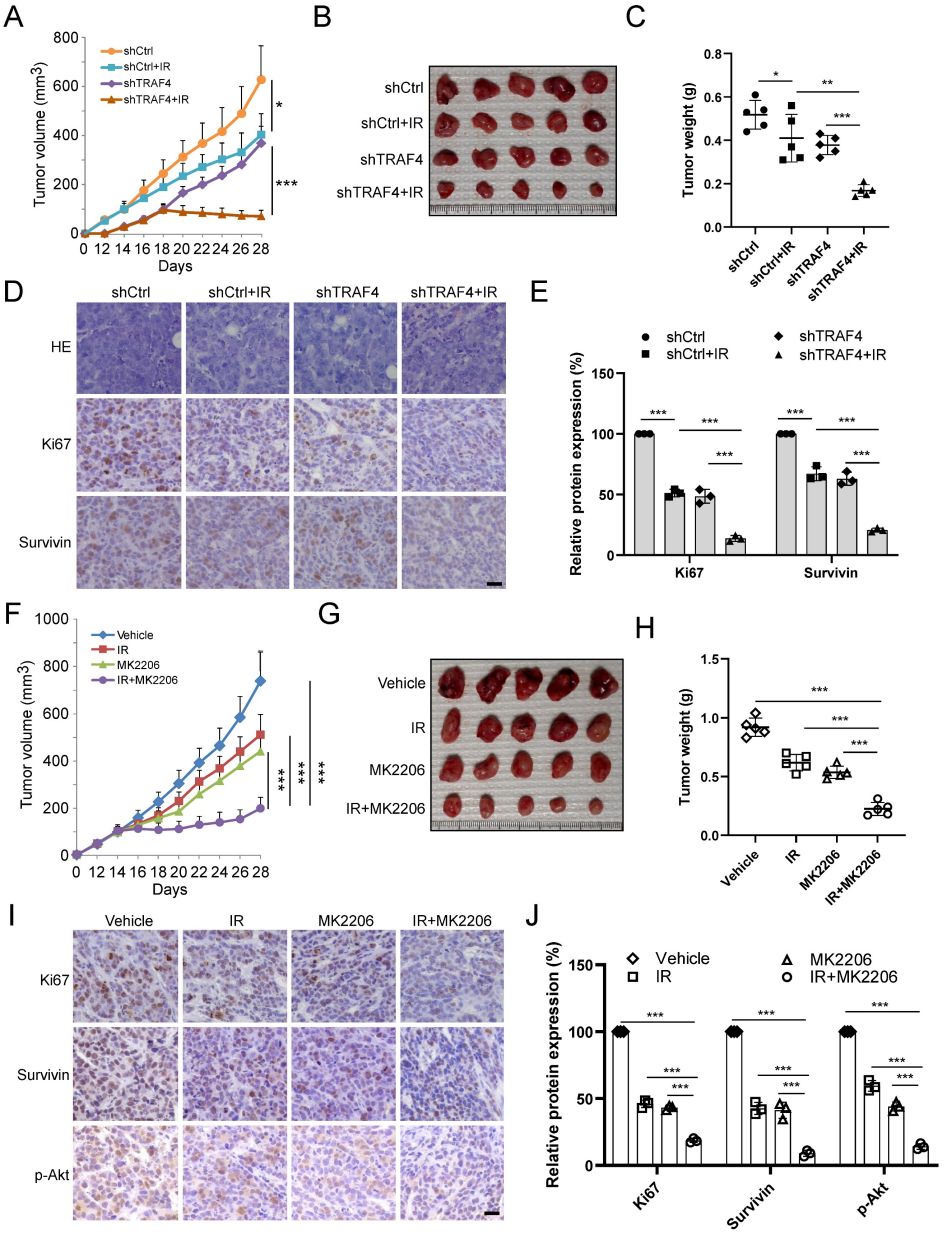
Knockdown TRAF4 sensitized NPC cells to IR treatment. (A-E) CNE2-derived xenograft tumors were treated without/with IR. Tumor volume (A), the images of tumor mass (B), and tumor weight (C) were recorded, and tumor tissues were subjected to IHC staining (D). *p < 0.05, **p < 0.01, ***p < 0.001. (E) The qualification of (D). Scale bar, 25 μm. ***p < 0.001. (F-I) MK2206 enhanced IR-induced tumor suppression *in vivo*. The volume (F), mass (G), and weight (H) of CNE2 tumors treated with vehicle, IR, MK2206, or IR and MK2206 combination. ***p < 0.001. Tumor tissues were subjected to IHC staining (I). (J) Qualification of (I). Scale bar, 25 μm. ***p < 0.001.

To further determine the effect of TRAF4 on IR *in vivo*, the xenograft tumor model was generated using TRAF4 knockdown CNE2 stable cell lines. The *in vivo* tumor growth was delayed in the TRAF4-deficient CNE2 tumors after IR (2 Gy) treatment three times (Total 6 Gy) compared with that in TRAF4-proficient CNE2 tumors (Fig. 6A). Moreover, the tumor mass (Fig. 6B) and tumor weight (Fig. 6C) of TRAF4-depleted CNE2 tumors after IR treatment were prominently reduced consistently. IHC staining showed that knockdown of TRAF4 synergized with IR to reduce survivin and Ki67 expression (Fig. 6D-E). To investigate the effect of Akt signaling on survivin expression and IR sensitivity, the *in vivo* tumor model was conducted with Akt inhibitor MK2206. As shown in Fig. 6F-H, the growth (Fig. 6F), mass (Fig. 6G), and weight (Fig. 6H) of CNE2-derived tumors were suppressed by either monotherapy of IR or MK2206 and were further enhanced by the combination. IHC staining results suggested that the expression level of Ki67, survivin, and Akt phosphorylation decreased after monotherapy or in combination, and combination therapy's impact was more significant (Fig. 6I-J). These results suggest that the *in vivo* tumor growth was suppressed significantly after targeting TRAF4/Akt axis.

### TRAF4 is positively correlated with survivin in NPC specimens

**Figure 7:**
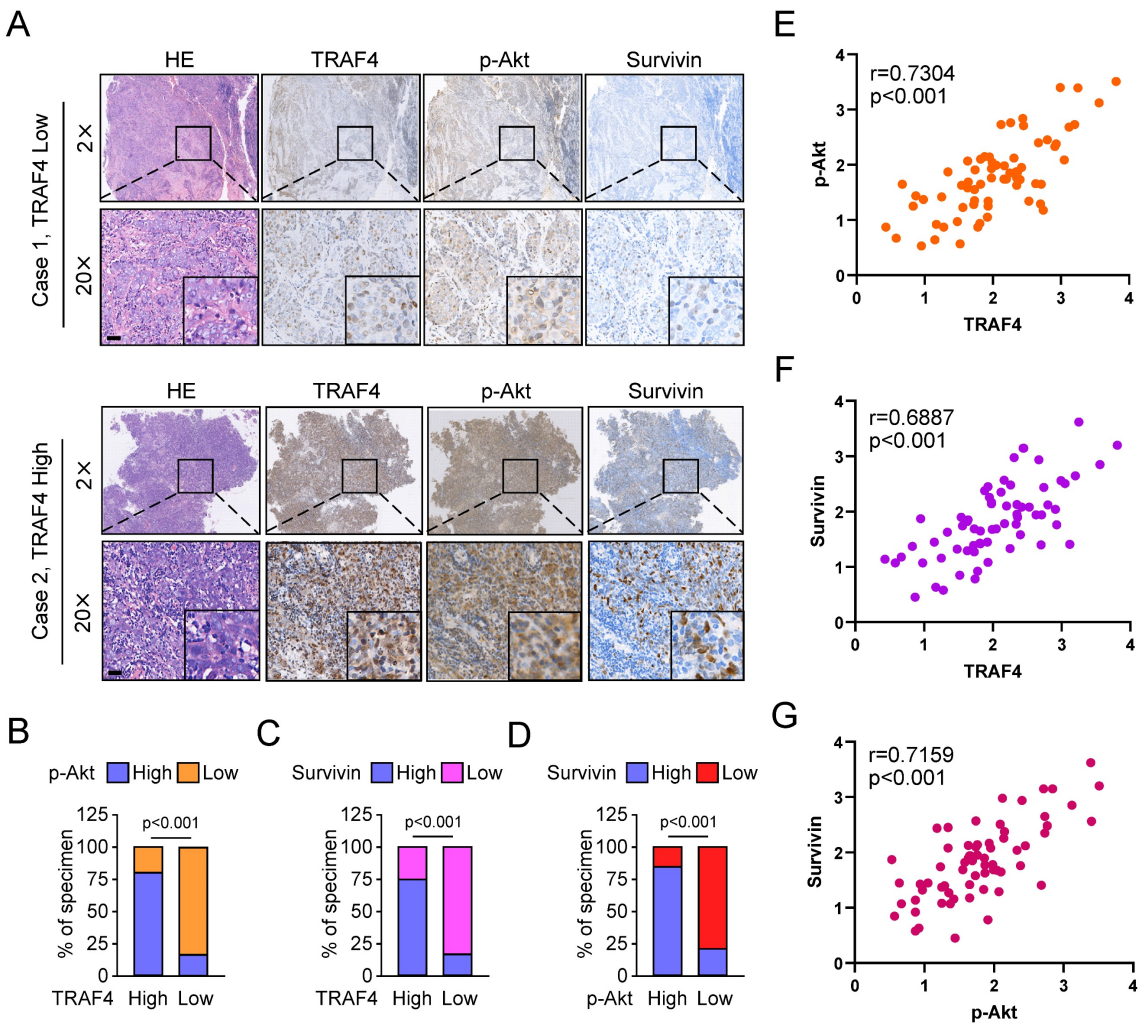
TRAF4 positively correlates with p-Akt and survivin in NPC specimens. (A) The representative IHC images with high or low TRAF4, p-Akt, and survivin from 67 cases of NPC samples. Scale bar, 40 μm. (B and C) The percentage of samples exhibiting high or low TRAF4 expression compared to p-Akt (B) and survivin (C) expression levels. (D) The percentage of specimens exhibiting high or low p-Akt expression compared to survivin expression. (E-G) The scatterplot indicated the positive correlation between p-Akt and TRAF4 (E), survivin and TRAF4 (F), and survivin and p-Akt (G) expression in tumor tissues expressing low or high TRAF4.

To demonstrate the clinical relevance of our findings, we examined TRAF4, p-Akt, and survivin expression in NPC tumor tissues. As shown in Fig. 7A, the representative images exhibited low or high TRAF4, p-Akt, and survivin expression levels in our cohort. Amongst 67 patients, 42 p-Akt high-expressing cases were seen in all 54 TRAF4 high-expressing individuals, 41 of 54 patients with TRAF4 high expression also exhibited upregulation of survivin protein level (Fig. 7B and C). Moreover, elevated activation of Akt was accompanied by overexpression of survivin, 38 survivin high-expressing cases were detected in all 44 p-Akt high-expressing patients (Fig. 7D). The expression of TRAF4, p-Akt, and survivin proteins was a positive correlation in NPC tumor tissues expressing low or high TRAF4 (Fig. 7E-G). These results revealed that TRAF4 and its downstream proteins p-Akt and survivin were overexpressed and positively correlated in NPC tissues.

### Overexpression of TRAF4 confers radioresistance

**Figure 8:**
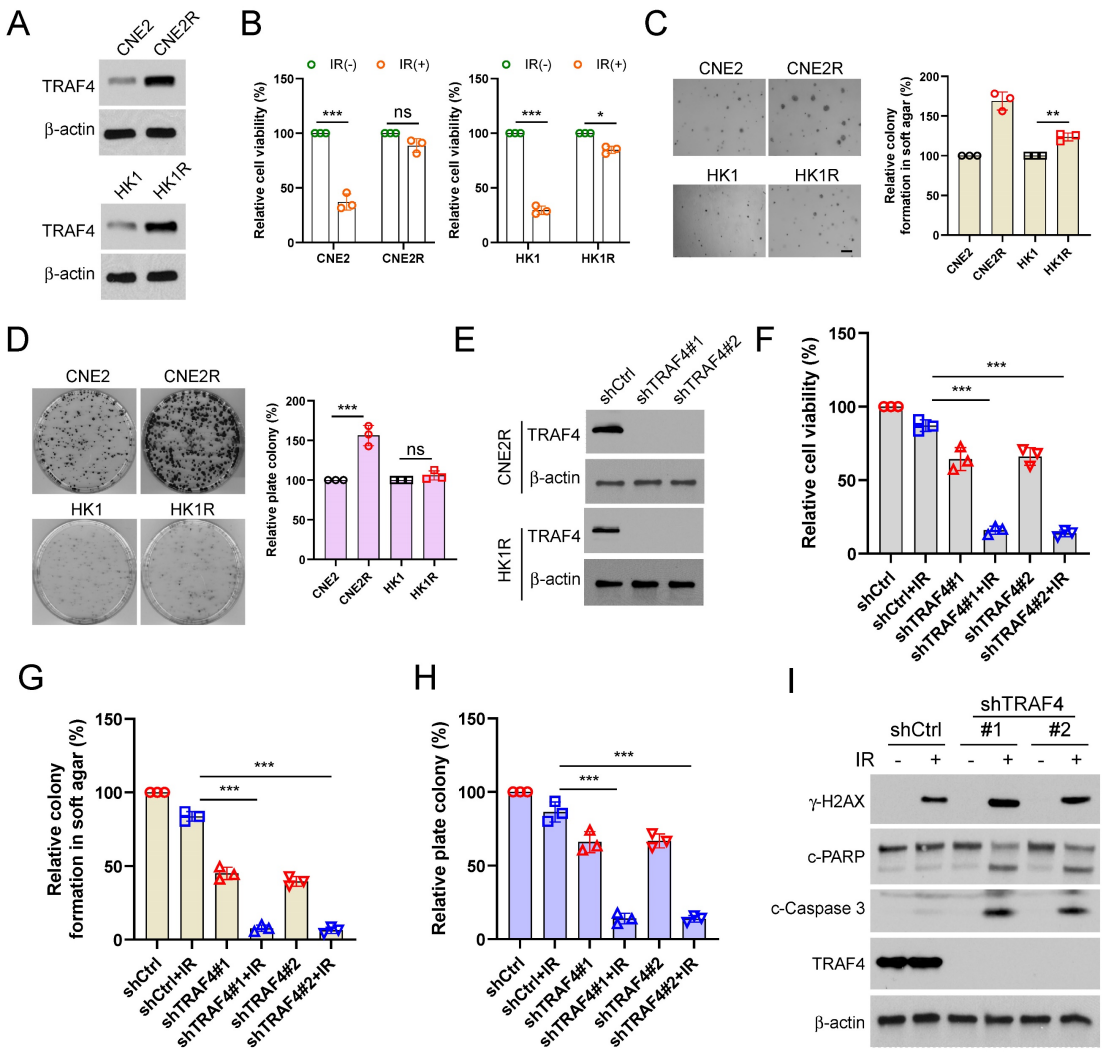
TRAF4 is vital for maintaining the radioresistant characteristics of NPC cells. (A) IB assay for TRAF4 expression in CNE2/CNE2R and HK1/HK1R cells. (B) The cell viability of CNE2/CNE2R and HK1/HK1R cells exposed to IR (4 Gy) was detected by MTS assay. ns: not statistically significant, *p < 0.05, ***p < 0.001. (C-D) Soft agar (C) and plate colony formation assay (D) of CNE2/CNE2R and HK1/HK1R cells. ns: not statistically significant, **p < 0.01, ***p < 0.001. (E) IB analysis of TRAF4 expression in TRAF4-depleted CNE2R and HK1R cells. (F-I) The shTRAF4-CNE2R cells were treated with/without IR (4 Gy) and maintained for 48 h. Cell viability was tested by MTS assay (F). ***p < 0.001. The anchorage-independent cell growth was evaluated using soft agar assay (G), and the anchorage-dependent growth was assessed by plate colony formation assay (H). ***p < 0.001. Apoptosis and DNA damage were tested by IB analysis (I).

Our current studies showed that TRAF4 was associated with radiotherapy sensitivity. Hence, we detected TRAF4 protein levels in two pairs of acquired radioresistant NPC cells, CNE2/CNE2R and HK1/HK1R. The IB data showed that TRAF4 was highly overexpressed in radioresistant CNE2R and HK1R when compared to parental CNE2 and HK1 cells, respectively (Fig. 8A). The viability of CNE2R and HK1R cells was unaffected following 4 Gy IR treatment. In contrast, the viability of parental CEN2 and HK1 cells was reduced significantly (Fig. 8B). The radioresistant CNE2R and HK1R cells exhibited a stronger colony formation ability in soft agar (Fig. 8C). Furthermore, the plate colony formation assay revealed that the CNE2R cells formed greater colonies on the plate than CNE2 cells. However, no significant difference was present between HK1 and HK1R cells (Fig. 8D). CNE2R and HK1R cells with TRAF4 deficiency were further established (Fig. 8E). We found that knockdown of TRAF4 significantly inhibited the cell viability (Fig. 8F and S6A) and colonies formation in soft agar (Fig. 8G and S6B) or on plates (Fig. 8H and S6C) after IR treatment compared with shCtrl cells. Moreover, the IB results revealed that TRAF4 deficiency promoted IR-induced DNA damage, as the expression of γ-H2AX increased substantially (Fig. 8I). The cleaved-PARP, and -caspase 3, markers for apoptosis were elevated in TRAF4-depleted CNE2R cells with IR treatment (Fig. 8I), suggesting that knockdown of TRAF4 resensitized radiotherapy and triggered apoptosis in CNE2R cells.

To further determine whether the TRAF4-regulated Akt/Wee1/CDK1/Survivin axis plays a role in acquired radioresistance, we first established TRAF4-overexpressed CNE2 and HK1 cells and found that TRAF4 overexpression partially restored the cell viability of HK1 and CNE2 cells after IR treatment (Fig. S6D-F). Moreover, the ability of colonies formation in soft agar or on the plate in TRAF4-overexpressed HK1 or CNE2 cells after IR treatment was superior to the control group (Fig. S6G-N). Similarly, IR-induced apoptosis was suppressed as evidenced by the reduction of cleaved-caspase 3 proteins and caspase 3 activity in TRAF4-overexpressed CNE2 and HK1 cells (Fig. S6O-Q). These data demonstrated that the overexpression of TRAF4 facilitated radioresistance. Furthermore, we treated radioresistant CNE2R cells with Akt (MK2206), Wee1 (Adavosertib), or CDK1 (Flavopiridol) inhibitors in the presence or absence of irradiation. We found that the inhibitors could suppress the cell viability, and colony formations, and the inhibitory efficacy was further enhanced following IR treatment (Fig. S7A-C). As shown in Fig. S7D, gradually increased siSurvivin was transfected into CNE2R and HK1R cells. We then treated siSurvivin-CNE2R and HK1R cells with IR and discovered that the cell viability, colony formation on the plate or in soft agar was decreased in siSurvivin-CNE2R and -HK1R cells after IR treatment (Fig. S7E-J). Overall, these data suggested that TRAF4 is essential for maintaining the radioresistant characteristics of NPC cells.

**Figure 9:**
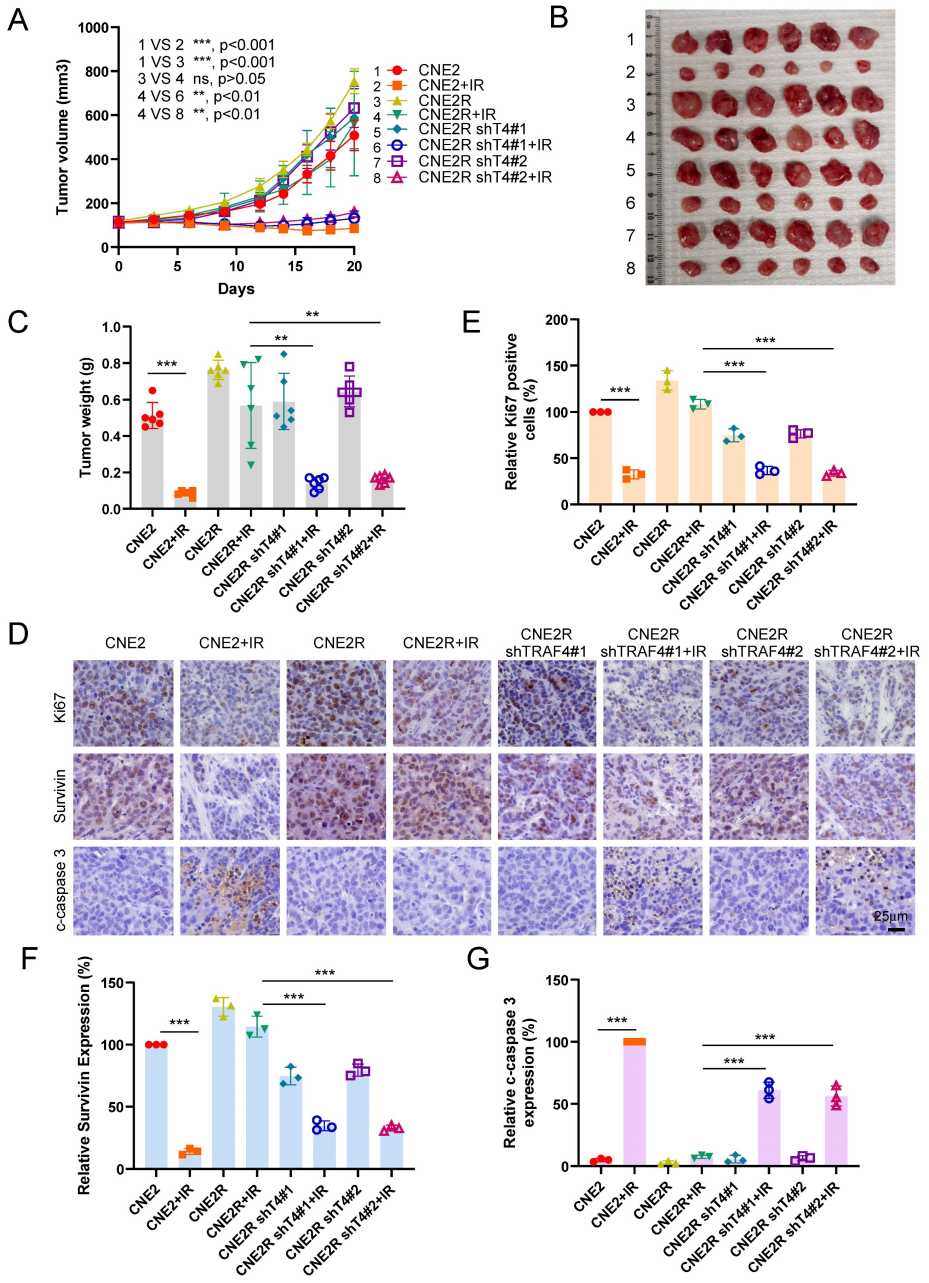
Knockdown of TRAF4 overcomes IR resistance *in vivo*. (A-D) CNE2, CNE2R-shCtrl, and shTRAF4 xenograft tumors were treated without/with IR. The tumor volume (A), the images of tumor mass (B), and tumor weight (C) were recorded. ns: not statistically significant, **p < 0.01, ***p < 0.001. Tumor tissues were subjected to IHC staining, and the representative images are shown in (D). Scale bar, 25 μm. (E-G) The qualification of Ki67 positive cells (E), survivin (F), and cleaved-caspase 3 (G) expression. Scale bar, 25 μm. ***p < 0.001.

The xenograft tumor model was further conducted to validate the effect of TRAF4 on radioresistance *in vivo*. The resistant CNE2R cells exhibited a stronger *in vivo* tumorigenic activity than the parental CNE2 cells (Fig. 9A-9C). IR (4 Gy) treatment for three times (Total 12 Gy) markedly inhibited tumorigenesis of parental CNE2 cells. However, the *in vivo* tumor development of radioresistant CNE2R cells was unchanged (Fig. 9A). Knockdown of TRAF4 resensitized CNE2R cells to radiotherapy, as the tumor volume, tumor mass, and tumor weight were reduced significantly when compared to that of the TRAF4 proficient CNE2R tumors (Fig. 9A-9C). IHC staining revealed that the expression of Ki67 and survivin were decreased in IR-treated TRAF4-deficient tumors, whereas the apoptosis marker, cleaved-caspase 3, was upregulated (Fig. 9D-9G). These results suggest that the knockdown of TRAF4 overcomes radioresistance in NPC cells.

## Discussion

Radiotherapy plays a vital role in treating nasopharyngeal carcinoma 4. Despite technological advances, the prognosis of NPC patients after radiotherapy varies widely 39, possibly related to the development of radioresistance 40. Previous studies have revealed that radiotherapy resistance is associated with autophagy dysfunction, tumor stem cells, and DNA damage response in NPC 41-43. Currently, several predictive markers of radioresistance in NPC have been identified, including β-lactamase-like-protein 2 44, CD38 45, homeobox A1 46, rab1A 47, fat mass and obesity-associated protein 48, and chloride intracellular channel 4 49. However, no promising reactive biomarkers can be used for clinics to improve radiosensitivity in NPC. A trustworthy radiosensitivity predictive marker can guide individualized treatment strategy with advantages. However, the ubiquitin-related predictors in NPC, especially in radioresistant NPC, remain unclear. Therefore, further studies are needed to explore biomarkers and potential mechanisms associated with radioresistance to develop effective therapeutic strategies for patients with NPC. We reported here that TRAF4 was highly expressed in NPC (Fig. 1) and radioresistant NPC cell lines (Fig. 8A). TRAF4 depletion overcomes radioresistant both *in vitro* and *in vivo*, suggesting that TRAF4 is a potential marker to predict radiosensitivity and a potential therapeutic target for NPC treatment.

Recently study showed that TRAF4-mediated atypical ubiquitination of SET domain bifurcated 1 induced the activation of the AKT pathway to facilitate glioblastoma proliferation 50. TRAF4 facilitates the ubiquitination of JNK1/2 and activates the JNK/c-Jun pathway to drive radioresistance in colorectal cancer 10. Moreover, TRAF4 catalyzes CHK1 ubiquitination and promotes DNA damage response to confer chemoresistance 16. In the current study, we explored how TRAF4 regulates the radiosensitivity of NPC cells and found that TRAF4 substantially regulates the proliferation and colony formation of NPC cells (Fig. 1). Our previous studies have shown that TRAF4-mediated K63-linked polyubiquitination of Akt, resulting EGF-induced Akt activation 32, 36. We report that TRAF4 deficiency decreased Akt ubiquitination, blocked the Akt/Wee1/CDK1/Survivin pathway, and increased the protein level of γ-H2AX and DNA damage. Knockdown TRAF4 promotes the radiosensitivity of NPC cells and activates apoptosis. All these results indicate that TRAF4 is a potential therapeutic target to overcome radioresistance in NPC treatment. Further studies are needed to explore the potential combinatorial effects of TRAF4 deficiency with other therapeutic approaches.

Increasing research showed that abnormally high survivin expression correlates with therapeutic resistance, significantly contributing to cancer recurrence or persistence 51. For example, paclitaxel resistance of NPC was associated with survivin upregulation caused by RSF1-mediated NF-κB signaling activation 52. Activation of CXCL12/CXCR4 confers radioresistance of colorectal cancer cells by upregulating survivin expression 53. Survivin correlates with chemotherapy resistance and plays an essential role in cell cycle progression, apoptosis, proliferation, and tumor formation and development of NPC 24, 26, 54. Survivin expression is tightly regulated by multiple manners. Survivin is regulated by p53, runt-related transcription factor 1, specificity protein 1, TGF-β, and HIF-1α at the transcriptional level 55, 56. Furthermore, survivin expression has been reported to be regulated by NF-κB, MAPK/ERK, and JAK/STAT signaling pathways 57, 58. Survivin is also controlled by post-translational modification, especially ubiquitination, which regulates tumor inhibition and promotion pathways. Previous studies have reported that some ubiquitinases or deubiquitinases such as FBXL7 59, XIAP 60, USP19 23, TRABID 61, and USP35 62 directly combined with survivin to regulate its stabilization and functions. The Thr34 phosphorylation stabilizes and prevents degradation of survivin caused by E3 ligase 33. However, the impact of TRAF4 on survivin in NPC remains elusive. Our data indicated that high-expressing survivin was positively correlated with TRAF4 in NPC tissues (Fig. 7). TRAF4 maintained survivin expression and inhibited survivin degradation in NPC cells. Moreover, we found that TRAF4 activated Akt/Wee1 axis is required for survivin Thr34 phosphorylation, which impaired FBXL7-mediated survivin ubiquitination and degradation. Previous study also demonstrated that survivin also regulated Wee1 protein degradation and CDK1 phosphorylation 37. However, our study indicated that total Wee1 and CDK1 proteins was unchanged (Fig. 5D), suggesting that survivin was not the upstream regulator. Additionally, silencing Wee1 or inhibiting CDK1 reduced the expression of survivin and promoted radiosensitization (Fig. 5D, S5A-B and S7A-C). The results further clarified that Wee1 and CDK1 were upstream regulator of survivin. Therefore, promoting the degradation of survivin or inhibiting its expression is a promising strategy for radiosensitization.

Several inhibitors that target survivin have been developed recently. YM155 plays an anticancer role by directly suppressing survivin promoter or expression. However, previous studies showed that YM155 failed to exhibit an obvious benefit in phase I/II clinical trials 63. Terameprocol suppresses survivin expression and exhibits a robust antitumor effect in cervical intraepithelial neoplasia in a phase II study 56. 5-aminoimidazole-4-carboxamide ribonucleotide (AICAR), in the state of preclinical research, showed antiproliferative and proapoptotic activity in cancer by destabilizing survivin 64. In addition, other small-molecule survivin inhibitors, including FL118, SF002-96-1, WM-127, GDP366, UC-112, and PZ-6-QN, also exhibit antitumor effects but have not been applied to clinical practice 65. In recent years, natural products also exhibited excellent antitumor effects in preclinical models by targeting critical cellular signaling pathways associated with survivin. Dihydromyricetin suppressed EGFR signaling to inhibit survivin expression, resulting in the downregulation of survivin phosphorylation and the degradation of survivin induced by ubiquitination in non-small cell lung cancer cells 57. Ethanol extract from Resina Draconis targeted the METTL3-m6 A-Survivin axis to inhibit the growth of hepatocellular carcinoma cells 66. However, only preclinical studies on these inhibitors are insufficient, and more clinical validation is needed to determine the efficacy and safety of inhibitors at present.

## Conclusions

We reported that TRAF4 depletion attenuates survivin phosphorylation via inhibiting the Akt/Wee1/CDK1 axis, resulting in survivin degradation and apoptosis activation. Our findings discovered that TRAF4 plays a crucial role in promoting the radioresistance of NPC by activating Akt and stabilizing survivin. Thus, a combinatorial approach of targeting TRAF4 or its downstream molecule, such as Akt with IR, would elevate the therapeutic effect of irradiation on nasopharyngeal carcinoma, which provides novel insights into rational cancer treatment.

## Supplementary Material

Supplementary figures.

## Abbreviations

NPC: Nasopharyngeal carcinoma
TRAF4: Tumor necrosis factor receptor-associated factor 4
IR: Irradiation
IAP: Inhibitor of apoptosis
CHX: Cycloheximide
IB: Immunoblotting
Co-IP: Co-immunoprecipitation
RT: room temperature
IF: Immunofluorescence
IHC: Immunohistochemical
NEM: N-ethylmaleimide
shRNA: short hairpin RNA
siRNA: short interfering RNA
WCE: whole cell extract
