# Supplementary material for: TRAF4 regulates ubiquitination-modulated survivin turnover and confers radioresistance
Source: Int J Biol Sci. 2024 Jan 1;20(1):182–99. doi: 10.7150/ijbs.87180 (PMC10750280; doi:10.7150/ijbs.87180)
Supplement: Supplementary file 1 — Supplementary figures. [file ijbsv20p0182s1.pdf]

Figure S1

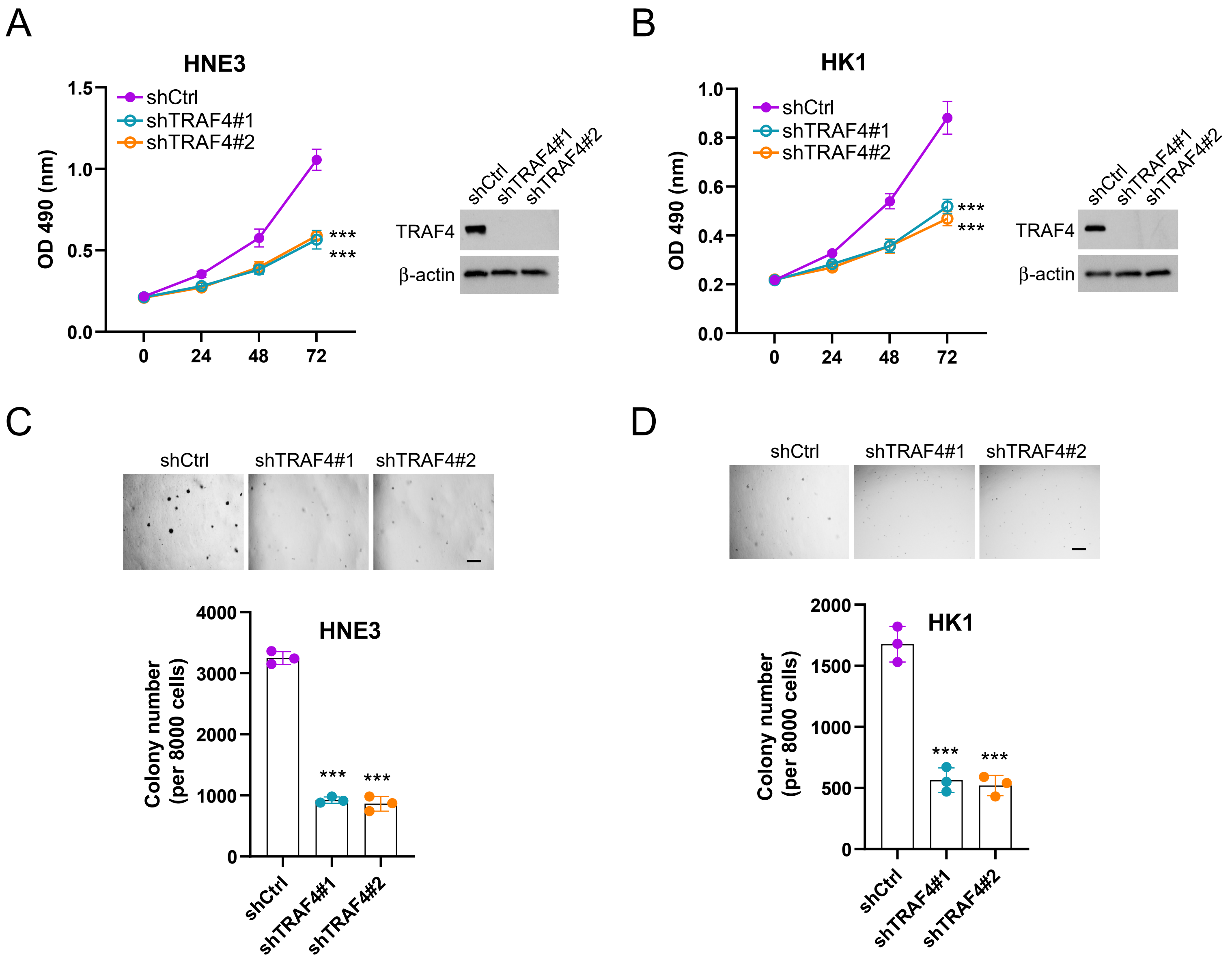

A
HNE3
OD 490 (nm)
shCtrl
shTRAF4#1
shTRAF4#2
***
***
0
24
48
72
TRAF4
β-actin
B
HK1
C
shCtrl
shTRAF4#1
shTRAF4#2
HNE3
Colony number (per 8000 cells)
D
HK1

Figure S2

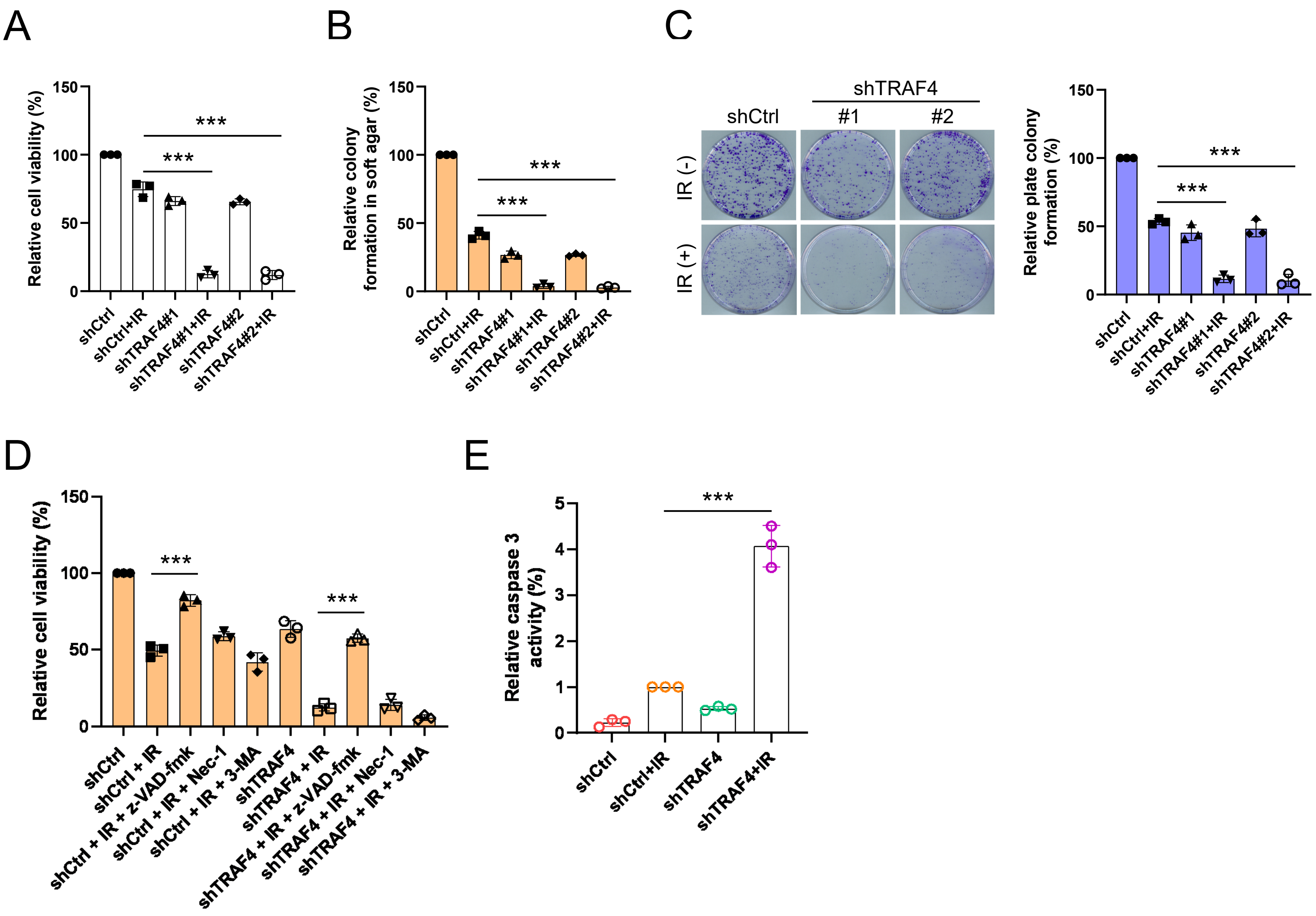
A
Relative cell viability (%)
150
100
50
0
***
***
shCtrl
shCtrl+IR
shTRAF4#1
shTRAF4#1+IR
shTRAF4#2
shTRAF4#2+IR
B
Relative colony formation in soft agar (%)
150
100
50
0
***
***
shCtrl
shCtrl+IR
shTRAF4#1
shTRAF4#1+IR
shTRAF4#2
shTRAF4#2+IR
C
shTRAF4
shCtrl
#1
#2
IR (-)
IR (+)
Relative plate colony formation (%)
150
100
50
0
***
***
shCtrl
shCtrl+IR
shTRAF4#1
shTRAF4#1+IR
shTRAF4#2
shTRAF4#2+IR
D
Relative cell viability (%)
150
100
50
0
***
***
shCtrl
shCtrl + IR
shCtrl + IR + z-VAD-fmk
shCtrl + IR + Nec-1
shCtrl + IR + 3-MA
shTRAF4
shTRAF4 + IR
shTRAF4 + IR + z-VAD-fmk
shTRAF4 + IR + Nec-1
shTRAF4 + IR + 3-MA
E
Relative caspase 3 activity (%)
5
4
3
2
1
0
***
shCtrl
shCtrl+IR
shTRAF4
shTRAF4+IR

Figure S3

A

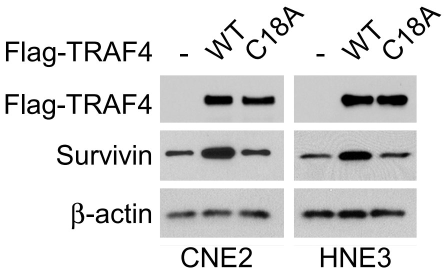

Flag-TRAF4
-
WT
C18A
-
WT
C18A
Flag-TRAF4
Survivin
β-actin
CNE2
HNE3

Figure S4

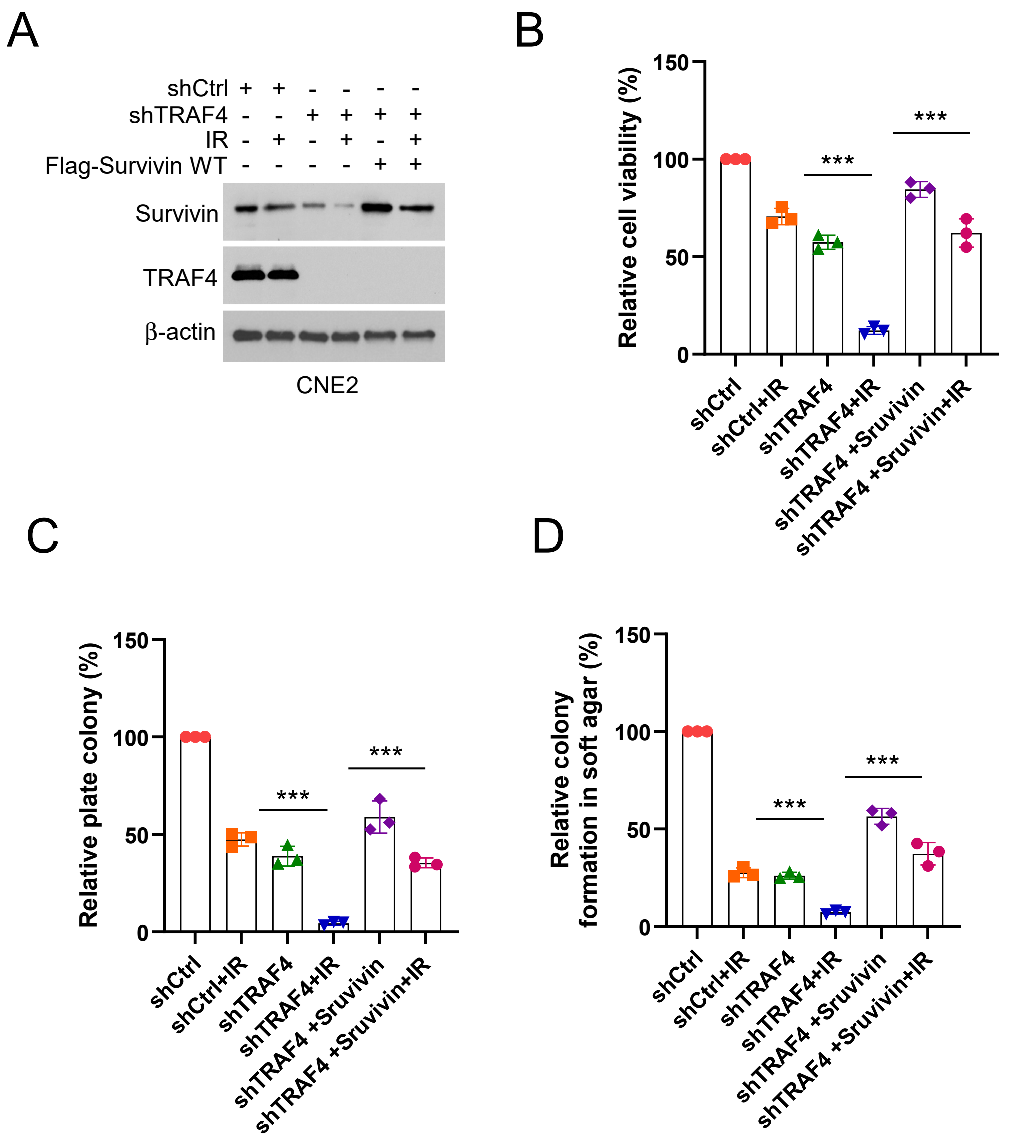
A
shCtrl + + - - - -
shTRAF4 - - + + + +
IR - + - + - +
Flag-Survivin WT - - - - + +
Survivin
TRAF4
β-actin
CNE2
B
Relative cell viability (%)
150
100
50
0
***
***
shCtrl
shCtrl+IR
shTRAF4
shTRAF4+IR
shTRAF4 +Sruvivin
shTRAF4 +Sruvivin+IR
C
Relative plate colony (%)
150
100
50
0
***
***
shCtrl
shCtrl+IR
shTRAF4
shTRAF4+IR
shTRAF4 +Sruvivin
shTRAF4 +Sruvivin+IR
D
Relative colony
formation in soft agar (%)
150
100
50
0
***
***
shCtrl
shCtrl+IR
shTRAF4
shTRAF4+IR
shTRAF4 +Sruvivin
shTRAF4 +Sruvivin+IR

Figure S5

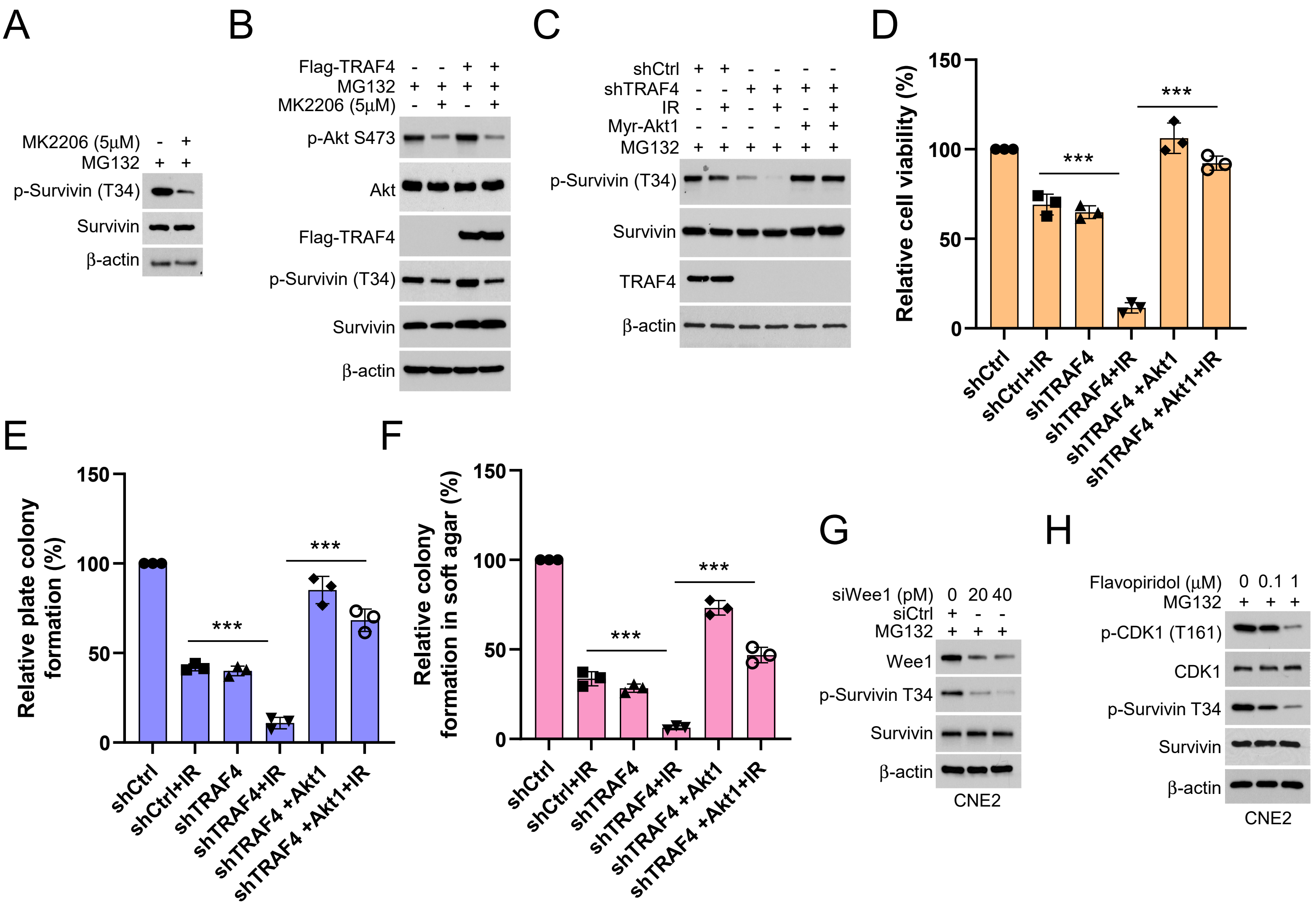
A
MK2206 (5μM) - +
MG132 + +
p-Survivin (T34)
Survivin
β-actin
B
Flag-TRAF4 - - + +
MG132 + + + +
MK2206 (5μM) - + - +
p-Akt S473
Akt
Flag-TRAF4
p-Survivin (T34)
Survivin
β-actin
C
shCtrl + + - - - -
shTRAF4 - - + + + +
IR - + - + - +
Myr-Akt1 - - - - + +
MG132 + + + + + +
p-Survivin (T34)
Survivin
TRAF4
β-actin
D
Relative cell viability (%)
150
100
50
0
***
***
shCtrl
shCtrl+IR
shTRAF4
shTRAF4+IR
shTRAF4 +Akt1
shTRAF4 +Akt1+IR
E
Relative plate colony formation (%)
150
100
50
0
***
***
shCtrl
shCtrl+IR
shTRAF4
shTRAF4+IR
shTRAF4 +Akt1
shTRAF4 +Akt1+IR
F
Relative colony formation in soft agar (%)
150
100
50
0
***
***
shCtrl
shCtrl+IR
shTRAF4
shTRAF4+IR
shTRAF4 +Akt1
shTRAF4 +Akt1+IR
G
siWee1 (pM) 0 20 40
siCtrl + - -
MG132 + + +
Wee1
p-Survivin T34
Survivin
β-actin
CNE2
H
Flavopiridol (μM) 0 0.1 1
MG132 + + +
p-CDK1 (T161)
CDK1
p-Survivin T34
Survivin
β-actin
CNE2

Figure S6

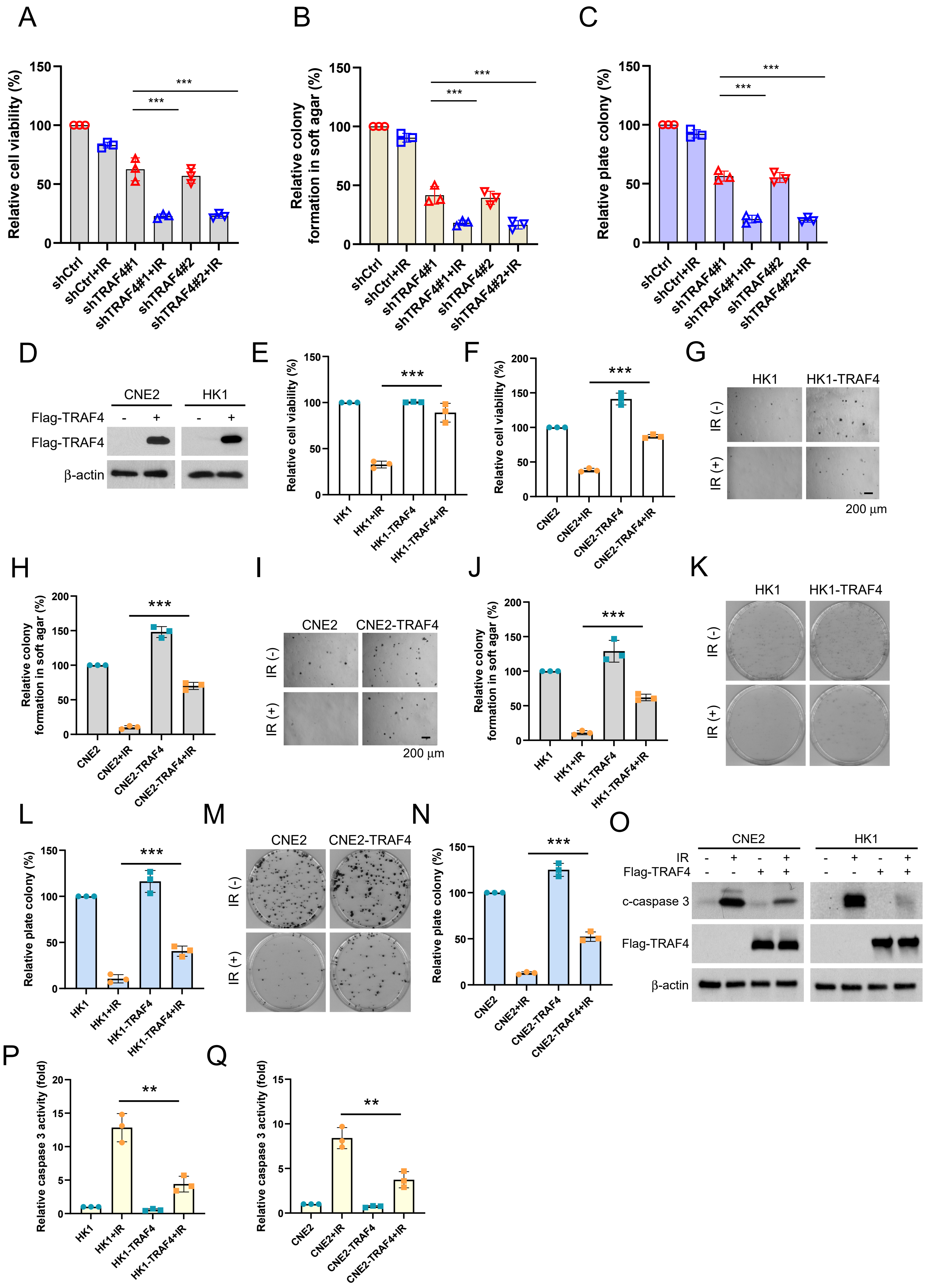
A
B
C
D
E
F
G
H
I
J
K
L
M
N
O
P
Q
Relative cell viability (%)
Relative colony formation in soft agar (%)
Relative plate colony (%)
shCtrl
shCtrl+IR
shTRAF4#1
shTRAF4#1+IR
shTRAF4#2
shTRAF4#2+IR
CNE2
HK1
Flag-TRAF4
β-actin
HK1-TRAF4
CNE2-TRAF4
IR (-)
IR (+)
200 µm
IR
c-caspase 3
Relative caspase 3 activity (fold)
HK1+IR
HK1-TRAF4+IR
CNE2+IR
CNE2-TRAF4+IR

Figure S7

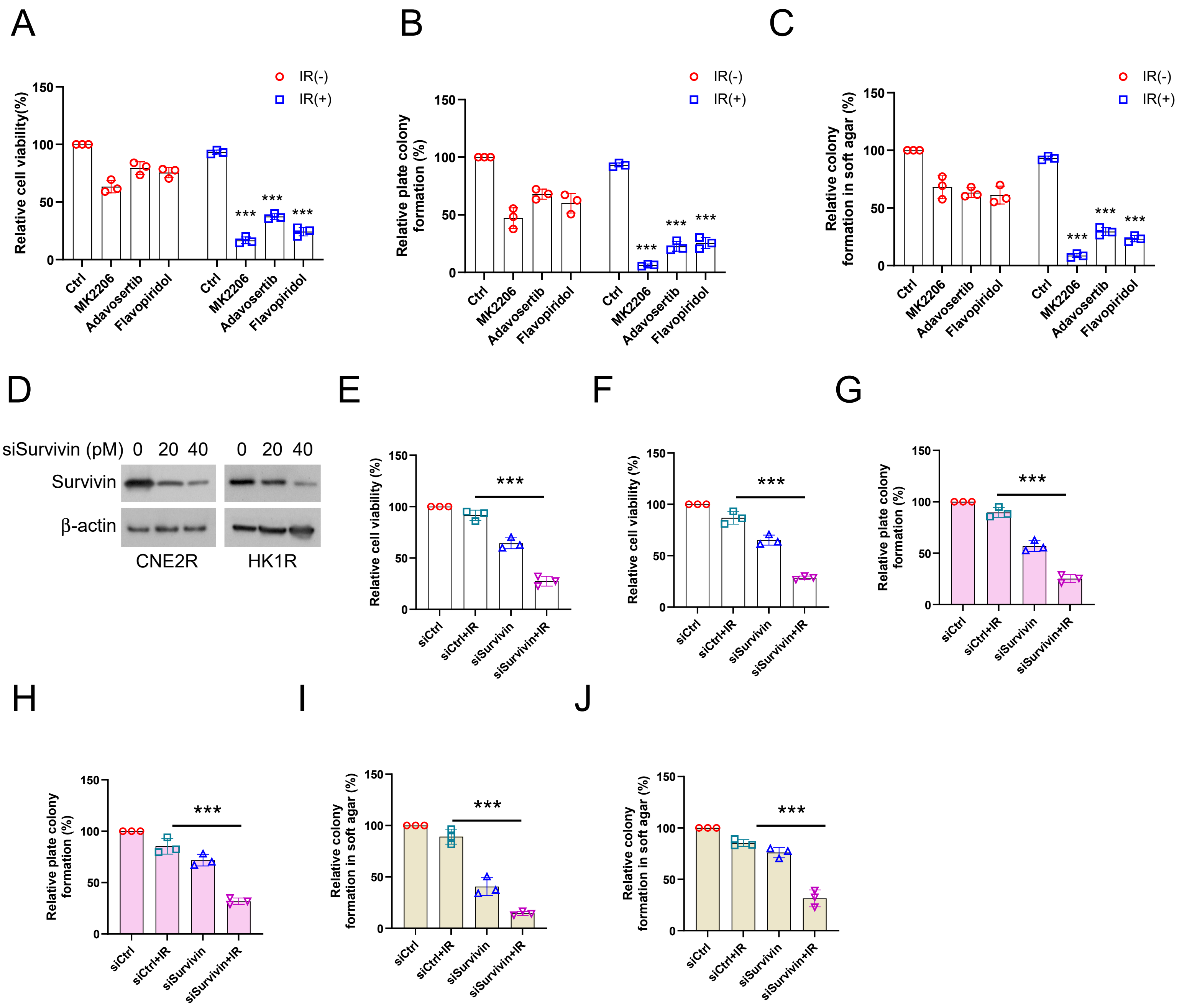
A
B
C
IR(-)
IR(+)
Relative cell viability(%)
Relative plate colony formation (%)
Relative colony formation in soft agar (%)
Ctrl
MK2206
Adavosertib
Flavopiridol
***
D
siSurvivin (pM) 0 20 40 0 20 40
Survivin
β-actin
CNE2R
HK1R
E
F
G
H
I
J
Relative cell viability (%)
Relative plate colony formation (%)
Relative colony formation in soft agar (%)
siCtrl
siCtrl+IR
siSurvivin
siSurvivin+IR
***
0
50
100
150

Supplementary Figure 1: (A-B) MTS assay (left) was performed to detect cell viability. TRAF4 expression was validated by IB assay (right) in HNE3 (A) and HK1 (B) cells with TRAF4 depletion. ***$p < 0.001$. (C-D) Soft agar assay analysis of colony formation of HNE3 (C) and HK1 (D) cells with TRAF4 knockdown. Top, representative images; bottom, qualification. Scale bar 200 μm. ***$p < 0.001$.

Supplementary Figure 2: (A-C) TRAF4-depleted HNE3 cells were treated without/with IR (2 Gy). (A) The cell viability was tested by MTS assay after IR treatment for 24 h. ***$p < 0.001$. (B) Soft agar assay analysis of colony formation in shTRAF4 HNE3 cells. ***$p < 0.001$. (C) Plate colony formation of shTRAF4 HNE3 cells. Left, representative images; right, qualification. ***$p < 0.001$. (D) HNE3 shTRAF4 cells were pretreated with pan-caspase inhibitors such as z-VAD-fmk, necrostatin-1 (Nec-1), and 3-MA for 4 h, followed by IR (4 Gy) treatment and maintained for 24 h. MTS analysis was used to test cell viability. ***$p < 0.001$. (E) HNE3 shTRAF4 cells were exposed to IR (4 Gy) and maintained for 48 h. The caspase 3 activity was detected by the caspase 3 assay Kit. ***$p < 0.001$.

Supplementary Figure 3: IB analysis of indicated protein expression in vector or Flag TRAF4 WT- or C18A mutant-overexpressed CNE2 and HNE3 cells.

Supplementary Figure 4: (A-D) Flag-Survivin WT was transfected into shTRAF4-CNE2 cells for 24 h, followed by irradiation (2 Gy) and cultured for 24 h. WCE was subjected to IB analysis (A), cell viability was examined by MTS assay (B), and colony formation was detected by plate colony formation (C) and soft agar assay (D). ***$p < 0.001$.

Supplementary Figure 5: (A) CNE2 cells were treated with MK2206 for 24 h followed by MG132 treated for 6 h. The WCE was harvested and subjected to IB assay. (B) Flag-TRAF4 was transfected into CNE2 cells for 24 h, and then the cells were treated with MK2206 for 24 h by subsequent MG132 treatment for 6 h. The WCE was subjected to IB analysis. (C) Mry-Akt1 was transfected into shTRAF4-CNE2 cells for 24 h, followed by irradiation (2 Gy) for 24 h and MG132 treatment for 6 h. The WCE was subjected to IB analysis. (D-F) Mry-Akt1 was transfected into TRAF4-depleted CNE2 cells for 24 h, followed by IR (2 Gy) treated for another 24 h, cell viability was tested by MTS assay (D), colony formation was examined by plate colony formation (E) and soft agar (F) assays. ***$p < 0.001$. (G) The gradually increased Wee1 siRNA was transfected into CNE2 cells, and the WCE was harvested to detect the indicated protein expression using IB assay. (H) IB analysis of CNE2 cells treated with the gradually increased Flavopiridol.

Supplementary Figure 6: (A-C) The shTRAF4-HK1R cells were treated with/without IR (4 Gy) and maintained for 24 h. Cell viability was measured using MTS assay (A). ***$p < 0.001$. The anchorage-independent cell growth was assessed by soft agar assay (B), and the anchorage-dependent growth was evaluated using plate colony formation

assay (C). ***$p < 0.001$. (D) IB analysis of TRAF4 expression in TRAF4-overexpressed CNE2 and HK1 cells. (E-Q) Flag-TRAF4 were transfected into HK1 and CNE2 cells for 24 h by subsequent irradiation (4 Gy) for 48 h. (E-F) MTS assay was performed to detect the cell viability. ***$p < 0.001$. (G-N) The anchorage-independent cell growth was evaluated using soft agar assay (G-J), and the anchorage-dependent growth was assessed by plate colony formation assay (K-N). Scale bar 200 μm. ***$p < 0.001$. O, IB analysis of cleaved-caspase 3 levels in TRAF4-overexpressed CNE2 and HK1 cells after IR treatment. P and Q, Caspase 3 activity of TRAF4-overexpressed HK1 and CNE2 cells with or without IR treatment was assessed using caspase 3 activity assay. **$p < 0.01$.

Supplementary Figure 7: (A-C) CNE2R cells were treated with various inhibitors, including MK2206, Adavosertib, and Flavopiridol. Cell viability was tested by MTS assay (A). ***$p < 0.001$. The anchorage-dependent growth was evaluated using plate colony formation assay (B), and the anchorage-independent cell growth was assessed using soft agar assay (C). ***$p < 0.001$. (D) IB analysis of Survivin levels in CNE2R and HK1R cells transfected with the gradually increased Survivin siRNA. (E-J) The siSurvivin or siCtrl were transfected into CNE2R (E, G, I) and HK1R (F, H, J) cells for 24 h followed by IR (4 Gy) treatment. MTS assay was used to measure cell viability (E-F). ***$p < 0.001$. The anchorage-dependent growth was evaluated using plate colony formation assay (G-H), and the anchorage-independent cell growth was assessed using soft agar assay (I-J). ***$p < 0.001$.
